# IRF8-dependent molecular complexes control the Th9 transcriptional program

**DOI:** 10.1038/s41467-017-01070-w

**Published:** 2017-12-12

**Authors:** Etienne Humblin, Marion Thibaudin, Fanny Chalmin, Valentin Derangère, Emeric Limagne, Corentin Richard, Richard A. Flavell, Sandy Chevrier, Sylvain Ladoire, Hélène Berger, Romain Boidot, Lionel Apetoh, Frédérique Végran, François Ghiringhelli

**Affiliations:** 1 Univ. Bourgogne Franche-Comté, F-21000 Dijon, France; 2 Centre de Recherche INSERM LNC-UMR1231, F-21000 Dijon, France; 3 Platform of Transfer in Cancer Biology, Centre Georges-François Leclerc, F-21000 Dijon, France; 4 Department of Immunobiology, Yale University, School of Medicine, New Haven, CT 06510 USA; 5 Howard Hughes Medical Institute, Chevy Chase, MD 20815 USA; 6 Department of Biology and Pathology of Tumours, Centre Georges-François Leclerc, F-21000 Dijon, France; 7 Department of Medical Oncology, Centre Georges-François Leclerc, F-21000 Dijon, France

## Abstract

Interferon regulatory factors (IRF) have critical functions in lymphoid development and in immune response regulation. Although many studies have described the function of IRF4 in CD4^+^ T cells, few have focused on the IRF4 homologue, IRF8. Here, we show that IRF8 is required for Th9 differentiation in vitro and in vivo. IRF8 functions through a transcription factor complex consisting of IRF8, IRF4, PU.1 and BATF, which binds to DNA and boosts *Il9* transcription. By contrast, IRF8 deficiency promotes the expression of other genes such as *Il4*, as IRF8 dimerises with the transcriptional repressor ETV6 and inhibits *Il4* expression. In vivo, IRF8 is essential for the anti-tumour effects of Th9 cells in mouse melanoma models. Our results show that IRF8 complexes boost the Th9 program and repress *Il4* expression to modulate Th9 cell differentiation, thereby implicating IRF8 as a potential therapeutic target to affect Th9 responses in cancer therapy.

## Abstract (web summary)

Interferon regulatory factors IRF regulate lymphoid development, but the specific function of IRF8 in helper T-cell polarization is unclear. Here the authors show that IRF8 forms a complex with IRF4, PU.1 and BATF to modulate the Th9 transcription program and expression of IL-4 and IL-9.

## Introduction

IL-9-producing T-helper cells (Th9) are a subset of CD4^+^T cells with proinflammatory functions. Th9 cells arise from reprogrammed Th2 cells upon stimulation with transforming growth factor β (TGF-β). Th9 cells have been generated in vitro from mouse naive T cells after stimulation with TGF-β and interleukin 4 (IL-4) in the presence of T-cell receptor (TCR) signalling and costimulation^1,2^. Mouse and human Th9 cells secrete IL-9 and IL-21 and contribute to the development of autoimmunity in experimental allergic encephalomyelitis. Like Th2 cells, Th9 cells are involved in the development of allergic diseases, such as atopic dermatitis and allergic airway inflammation such as asthma^3,4^. In helminth infection, also involving type 2 immune responses, Th9 cells are essential for parasite eradication^5^. We and others have found that Th9 cells also exert an indirect anti-tumour effect resulting from secretion of IL-9 and IL-21^6–8^.

The transcriptional program of Th9 cells involves the transcription factors STAT6, GATA3, PU.1 and IRF4. TGF-β induces the activation of the SMAD pathway and expression of PU.1, which restrains Th2 polarisation. In the absence of PU.1, Th9 polarisation is impaired. Conversely, PU.1 overexpression in Th2 cells decreases IL-4, IL-5 and IL-13 secretion and promotes IL-9 production^9^. IRF4 is required for Th9, as well as for T-follicular helper (Tfh), Th2 and Th17 cell differentiation. PU.1 and IRF4 need a partner to bind to DNA. In Th9 cells, IRF4 cooperates with trabscription factors AP-1 (activator protein 1) and BATF to induce the transcriptional program^10^. By contrast, a PU.1 partner is not identified.

IRF8 is structurally closed to IRF4. IRF8 is an important regulator for macrophage, dendritic cells (DC) and B-cell development and function. Like IRF4, IRF8 also requires cooperative binding factors to regulate transcription. IRF8 forms a heterodimer with BATF and PU.1 in myeloid cells^11^. Interestingly, IRF8 can also act as a transcriptional repressor when associated with the ETV6 transcription repressor in macrophages^12^. Finally, IRF8 is implicated in Th17 and Treg cell differentiation^13–15^.

Here we show that IRF8 is essential for Th9 cell differentiation with a dual role. IRF8 cooperates with IRF4, PU.1 and BATF to induce IL-9 production, but also collaborates with ETV6 to suppress IL-4 secretion. Finally, the deficiency of IRF8 in Th9 cells impairs their anti-tumour properties.

## Results

### IRF8 deficiency impairs Th9 cell development in vitro

**Fig. 1:**
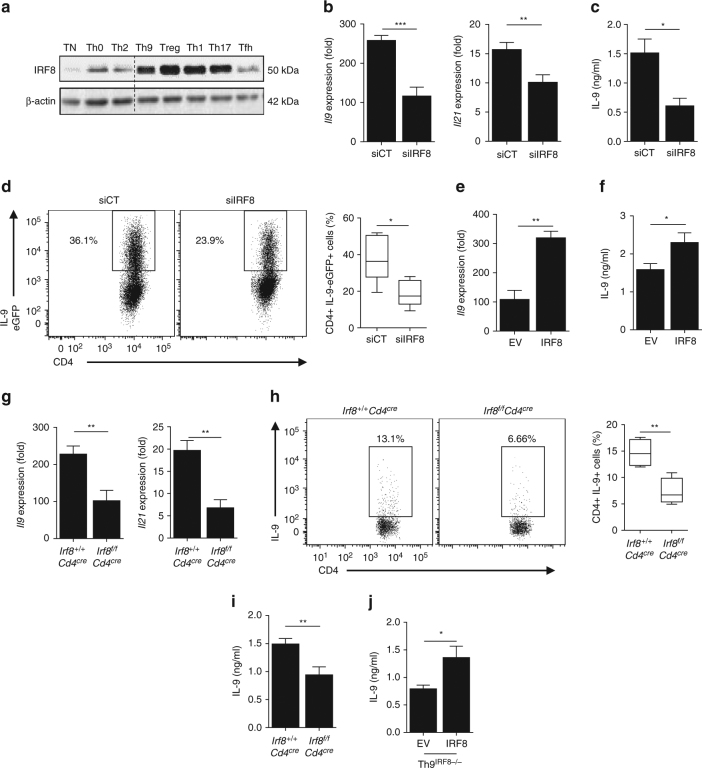
IRF8 deficiency impairs Th9 cell development in vitro. **a** Immunoblot analysis of IRF8 in WT naive CD4^+^ T cells or after 1 day of differentiation into Th0, Th2, Th9, Treg, Th1, Th17 and Tfh cells. **b**, **c** WT naive CD4^+^ T cells were transfected with control siRNA (siCT) or siRNA against *Irf8* (siIRF8), and then polarised under Th9 conditions. Relative expression of *Il9* and *Il21* mRNA (**b**) ELISA analysis of IL-9 protein in supernatant (**c**). **d** IL-9-eGFP naive CD4^+^ T cells were transfected with siCT or siIRF8, and then polarised under Th9 conditions. After 3 days of differentiation, eGFP-positive cells were assessed by flow cytometry (left: representative dot plot, right: means of four independent experiments). **e**, **f** WT naive CD4^+^ T cells were retrovirally infected with an empty-GFP vector (EV) or IRF8-GFP overexpressing vector (IRF8). GFP^+^ cells were sorted 2 days after infection and differentiated for 3 days into Th9 cells. *Il9* mRNA expression **e** and IL-9 protein release assessed with ELISA (**f**) were evaluated. **g**-**i**, *Irf8* ^*f/f*^ *Cd4* ^*cre*^ and *Irf8* ^*+/+*^ *Cd4* ^*cre*^ CD4^+^ T cells were differentiated into Th9 cells for 3 days. *Il9* and *Il21* mRNA expression (**g**) ELISA analysis of IL-9 protein in supernatant (**h**) and IL-9 intracellular staining in Th9 cells (**i**) (left: representative dot plot, right: means of four independent experiments). **j** IRF8-deficient naive CD4^+^ T cells were retroviral infected with an empty-GFP vector (EV) or IRF8-GFP overexpressing vector (IRF8). GFP^+^ cells were sorted 2 days after infection and differentiated for 3 days into Th9 cells. IL-9 protein release was evaluated by ELISA. ns, not significant; *P < 0.05, **P < 0.01; ***P < 0.001 (Mann–Whitney test). Data are from three **b**, **c**, **e**–**h** or two **j** independent experiments (mean and s.e.m.), **d**, **i** and one experiment representative of four independent experiments

First, we tested the expression level of IRF8 in the different subsets of in vitro differentiated helper T cells (Th). We observed that while IRF8 protein is almost absent in naive CD4 T cells, it is modestly expressed in Th0, Th2 and Follicular Helper T (Tfh) cells and strongly expressed in Th1, Th17, regulatory T cells (Treg) and Th9 cells (Fig. 1a).

To determine the function of interferon regulatory factor (IRF)8 in Th9 differentiation, we transfected naive CD4 T cells with *Irf8* SiRNA and induced in vitro Th9 differentiation. We checked that the SiRNA specifically reduced IRF8 expression at mRNA and protein levels without affecting IRF4 (Supplementary Fig. 1A). IRF8 depletion in Th9 cells was associated with reduced expression of Th9 cytokines *Il9* and *Il21* at the mRNA level and lower interleukin (IL)-9 secretion (Fig. 1b, c). Using CD4 from IL-9-eGFP (enhanced green fluorescent protein) transgenic mice, we observed a reduction in IL-9 production on a per cell basis in CD4^+^ T cells differentiated into Th9 cells in the presence of *Irf8* SiRNA (Fig. 1d). As a control, we observed that *Irf8* SiRNA did not induce transdifferentiation of Th9 cells as demonstrated by the absence of any induction of key transcription factors of Th1 (*Tbx21*), Th2 (*Gata3*), Th17 (*Rorc*) and Treg (*Foxp3*) as well Th1 and Th17 cell-related cytokines (IFN-γ and IL-17A, respectively). Conversely, IRF8 decreased Th2-related IL-4 production (Supplementary Fig. 1B, C). IRF8 is also expressed in other helper CD4 subsets (Supplementary Fig. 1E). We observed that *Irf8* SiRNA did not affect *Tbx21*, *Gata3*, *Foxp3* or *Rorc* expressions in Th1, Th2, Treg and Th17 cells, respectively but led to a small but significant increase in IL-17A production from Th17 as previously reported^16,17^ (Supplementary Fig. 1D, E). In contrast, infection of CD4^+^ T cells with IRF8-expressing retrovirus (Supplementary Fig. 1F) enhanced Th9 cell differentiation as demonstrated by increased production of IL-9 mRNA and protein (Fig. 1e, f) without inducing transdifferentiation (Supplementary Fig. 1G).

To validate these observations, we generated CD4-cre-*Irf8-*floxed mice (*Irf8* ^*f/f*^ *Cd4* ^*cre*^) which have elective invalidation of IRF8 in CD4^+^ and CD8^+^ T cells. These mice exhibit normal lymphoid tissues and normal distribution of immune cells in primary and secondary lymphoid organs. This deficiency does not impact the number and/or the development of T cells (Supplementary Fig. 2). We validated the construction by showing that Th9 cells generated in vitro from *Irf8* ^*f/f*^ *Cd4* ^*cre*^ mice expressed IRF4 protein while IRF8 was absent (Supplementary Fig. 1H). In vitro differentiated Th9 cells from *Irf8* ^*f/f*^ *Cd4* ^*cre*^ mice have reduced expression of Th9 cytokines such as *Il9* and *Il21* mRNA (Fig. 1g) and have also reduced capacity to produce IL-9 protein (Fig. 1h, i). In the same way as the results of Irf8 SiRNA, Th9 cells differentiated from *Irf8* ^*f/f*^ *Cd4* ^*cre*^ mice secrete more IL-4 than their control. IL-4 induction was not observed in Th1, Th2, Th17 and Treg cells differentiated from *Irf8* ^*f/f*^ *Cd4* ^*cre*^ mice (Supplementary Fig. 1J), thus demonstrating the specificity of the observations to Th9 cells. Restoring IRF8 expression in *Irf8* ^*f/f*^ *Cd4* ^*cre*^ T cells by IRF8-expressing retrovirus also restored IL-9 production by Th9 differentiated cells (Fig. 1j). Together these data highlight IRF8 participation in Th9 polarisation.

### IRF8 is induced through the SMAD3/TGF-β pathway

**Fig. 2:**
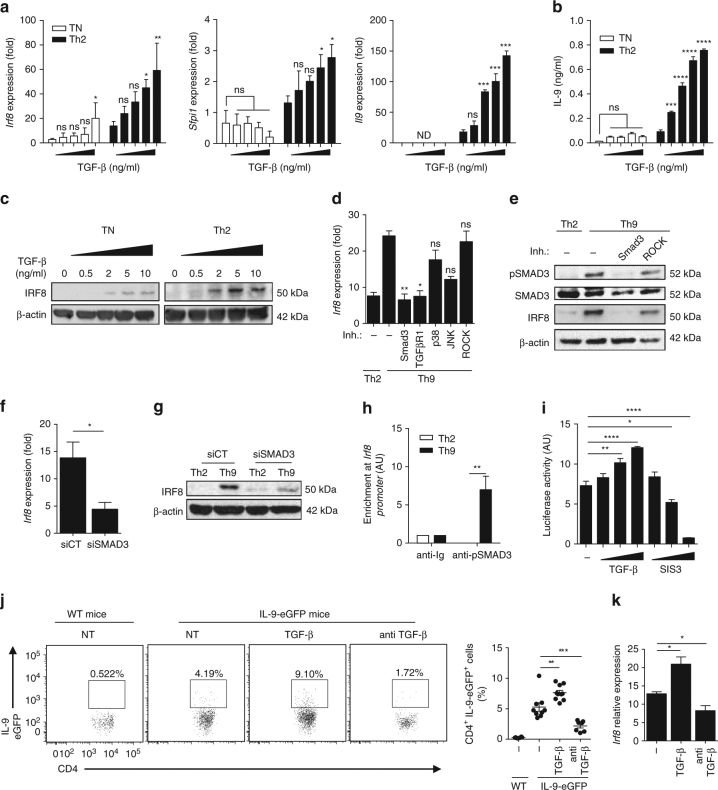
IRF8 is induced through the SMAD3/TGF-β pathway. **a**–**c** WT naive CD4^+^ cells (TN) without stimulation or Th2 cells were treated with increasing doses of TGF-β (0, 0.5, 2, 5 and 10 ng/ml). *Irf8, Sfpi1* and *Il9* mRNA expression after 16 h of treatment (**a**). ELISA analysis of IL-9 in supernatant after 3 days (**b**). Immunoblot analysis of IRF8 after 16 h of treatment (**c**). **d**, **e** WT naive CD4^+^ T cells (TN) were treated 1 h with pharmacological inhibitors against TGF-β signalling pathways (TGF-βR1, SMAD3, p38, ROCK, JNK). Cells were then polarised under Th9 conditions. *Irf8* mRNA expression in Th2 and Th9 cells or in treated Th9 cells after 24 h of treatment (**d**). Immunoblot analysis of IRF8, SMAD3 and phosphorylated SMAD3 (pSMAD3) in untreated Th2 and Th9 cells or in Th9 cells treated with SMAD3 or ROCK inhibitor after 24 h of treatment (**e**). **f**, **g** WT naive CD4^+^ T cells were transfected with siCT or siRNA against *Smad3* (siSMAD3), and polarised under Th2 or Th9 conditions. *Irf8* mRNA expression in Th9 cells after 24 h of treatment (**f**). Immunoblot analysis of IRF8 in Th2 or Th9 cells after 24 h of treatment. **h** ChIP analysis of the interaction between pSMAD3 and the *Irf8* promoter in Th2 and Th9 cells on the putative binding site at position −1275. **i** Transactivation of the *Irf8* promoter by TGF-β. Cells transfected with the *Irf8* promoter reporter plasmid were treated with increasing doses of TGF-β (25, 50, 100 ng/ml) or with increasing doses of SMAD3 inhibitor (SIS3) (150, 300, 600 nM). **j**, **k** B16F10 tumour-bearing IL-9-eGFP or WT mice were treated or not (NT) with TGF-β or anti-TGF-β. eGFP-positive cells were assessed in TILs by flow cytometry (left: representative dot plot, right: means of four independent experiments) **j** *Irf8* mRNA expression in eGFP-positive cells in TILs (**k**) ns, not significant; *P < 0.05, **P < 0.01; ***P < 0.001 (Mann–Whitney test (**f**), two-way ANOVA (**a**, **b**, **h**) or Kruskal–Wallis test (**d**, **i**–**k**)). Data are from three (**d**, **f**, **h**, **i**) or five **k** independent experiments (mean and s.e.m.), **j** and one experiment representative of five independent experiments

Kinetic study showed that *Irf8* mRNA is maintained throughout Th9 cell differentiation process while it is only transitionally expressed after TCR triggering during Th2 polarisation (Supplementary Fig. 3A). At the protein level, IRF8 expression is found in Th9 cells from 4 h of differentiation and this expression is maintained (Supplementary Fig. 3B). Th9 cells are generated in the presence of IL-4 and TGF-β and TCR triggering. As IRF8 expression in Th2 cells (generated with IL-4 and TCR triggering) is very weak and transient (Fig. 1a), we suspected that IRF8 induction and maintenance in Th9 cells depended on TGF-β. To test this hypothesis, we analysed *Irf8* mRNA expression in naive T cells and Th2 cells cultured with increasing doses of TGF-β. We observed that TGF-β induced *Irf8* mRNA expression in naive T cells without TCR stimulation. TGF-β also induced *Irf8* mRNA in Th2 cells in a dose-dependent manner (Fig. 2a). Similar results were obtained with Th0 cells generated by TCR triggering without polarising cytokines (Supplementary Fig. 3C). In contrast, TGF-β induced Th9 polarisation only in Th2 cells but not in Th0 cells as demonstrated with *Il9, Sfpi1* mRNA expression and IL-9 cytokine production (Fig. 2a, b and Supplementary Fig. 3D). These data suggest that TGF-β signalling alone, rather than the Th9 program, is involved in IRF8 expression. We confirmed this observation at the protein level. Indeed, TGF-β induced IRF8 protein expression in a dose-dependent manner in naive T cells, Th0 and Th2 (Fig. 2c and Supplementary Fig. 3E).

TGF-β is known to induce signalling via the canonical SMAD pathway or non-canonical pathways, which may involve Rho-associated protein kinase (ROCK), mitogen-activated-protein-kinase-activated p38 (p38 MAPK), c-Jun N-terminal kinase (JNK) and phosphoinositide 3-kinase. To identify the pathway involved in IRF8 induction, we used inhibitors of SMAD3, ROCK, p38 MAPK and JNK. We observed that only canonical SMAD pathway inhibition (TGFβ-R1 and SMAD3 inhibitor) could blunt *Irf8* mRNA and IRF8 protein expressions (Fig. 2d, e). To validate this observation, we used *Smad3* SiRNA (Supplementary Fig. 3F, G) and confirmed that its depletion reduced *Irf8* mRNA and protein expression in Th9 cells (Fig. 2f, g). Bioinformatics analysis revealed a putative SMAD3-binding site in the *Irf8* promoter at −1250 pb. SMAD3 immunoprecipitation showed that SMAD3 binds to the *Irf8* promoter in Th9 cells but not in Th2 cells (Fig. 2h). To investigate whether SMAD3 can directly induce *Irf8* promoter transactivation, we cloned the *Irf8* promoter (1300 base pairs) in a luciferase reporter plasmid and transfected NIH-3T3 cells. NIH-3T3 cells were then treated with either increasing doses of TGF-β to induce SMAD3 activation or increasing doses of SMAD3 inhibitor. TGF-β increased *Irf8* promoter transactivation while it was blunted by SMAD3 inhibitor (Fig. 2i).

Th9 cells were previously shown to control B16F10 melanomas growth^6,7^. Using IL-9-eGFP mice, we observed that, CD4 expressing IL-9-eGFP were present in B16F10 melanoma tumour bed. Blocking of TGF-β using monoclonal antibodies reduced both IL-9-eGFP^+^ CD4^+^ T cells and *Irf8* expression in CD4^+^ T cells. In contrast, injection of recombinant TGF-β into the tumour increased both IL-9-eGFP^+^ CD4^+^ T cells and *Irf8* expression in CD4^+^ T cells in vivo (Fig. 2j, k).

Together these data show that TGF-β is responsible for *Irf8* expression in Th9 cells in a SMAD3-dependent manner in vitro and in vivo.

### IRF8 is required for activation of the Th9 program

**Fig. 3:**
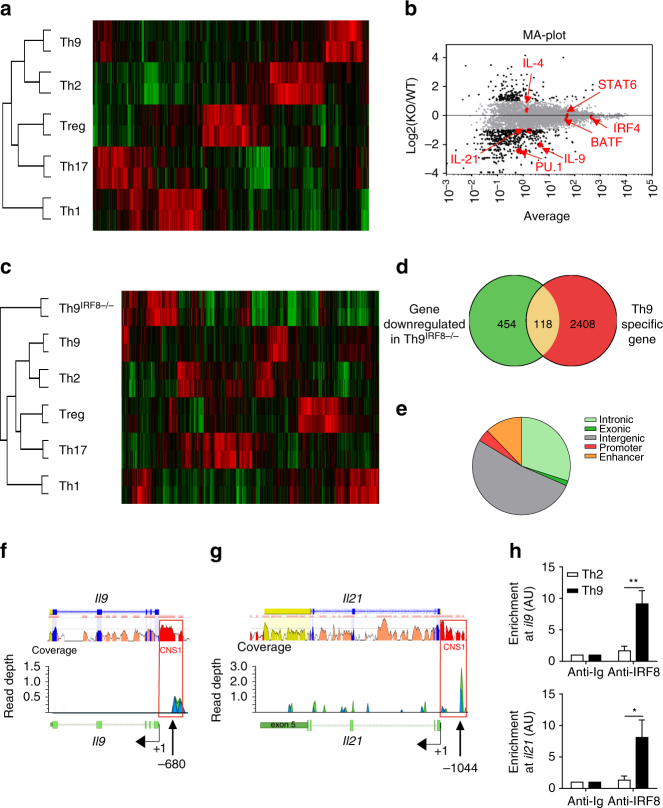
IRF8 is required for Th9 transcription program setting-up. **a** Heat map showing RNA sequencing data of WT Th1, Th2, Th9, Treg and Th17 cells differentiated for 24 h. **b** MA-plot showing RNA sequencing data of WT or IRF8^−/−^ Th9 cells after 24 h of differentiation. Genes upregulated or downregulated by at least 2-fold in IRF8^−/−^ Th9 cells compared with WT Th9 cells are labelled in black, genes with similar expression in both IRF8^−/−^ and WT Th9 cells are labelled in grey. Th9 cells-specific cytokines (*Il9* and *Il21*) and genes of interest (*Il4, Sfpi1, Batf, Irf4, Stat6*) are highlighted in red. **c** Heat map showing RNA sequencing data of WT Th1, Th2, Th9, Treg, Th17 and Th9^IRF8−/−^ cells differentiated for 24 h. **d** Venn diagram of genes downregulated in IRF8^−/−^ Th9 cells at 24 h (green) and Th9 cells-specific genes (red). **e** Distribution of IRF8 ChIP sequencing peaks in WT Th9 cells. **f**, **g** Top panel presents a screenshot from the ECR (evolutionary conserved regions) Browser web site of the mouse *Il9* (**f**) and *Il21* (**g**) genes. Exonic regions are in blue, intronic regions in pink, UTRs in yellow and CNS are in red. Bottom panel presents IRF8-binding peaks in WT Th9 cells at *Il9* (**f**) and *Il21* (**g**) loci. **h** ChIP analysis of the interaction between IRF8 and the CNS1 of *Il9* or *Il21* promoter in Th2 and Th9 cells. ns, not significant; *P < 0.05, **P < 0.01; ***P < 0.001 (two-way ANOVA (**h**)). Data are from four **h** independent experiments (mean and s.e.m.)

To further investigate the molecular explanation of IRF8 function during Th9 cell differentiation, we performed transcriptomic analysis of wild type (WT) Th1, Th2, Th9, Th17 and Treg cells at 24 and 48 h differentiation to determine the Th9 cell-specific genes (Fig. 3a and Supplementary Fig. 4A). We noted that 2526 genes were specifically overexpressed in Th9 cells at 24 h and 2122 at 48 h. When comparing transcriptomic profiles of WT and Th9^IRF8−/−^ cells, we observed that the expression of 572 genes was significantly reduced by at least 2-fold at 24 h and 217 genes were induced (Fig. 3b). Similarly, we observed that the expression of 582 genes was significantly reduced by at least 2-fold at 48 h and 308 genes were induced (Supplementary Fig. 4B), suggesting that IRF8 is important throughout along Th9 cell differentiation. Interestingly, a dendrogram and a hierarchical clustering heat map indicated that Th9^IRF8−/−^ cells did not transdifferentiate to another CD4 subset (Fig. 3c). We observed that 20.62% at 24 h and 19.81% at 48 h of IRF8-dependent genes were Th9 cell-specific genes, suggesting a major function of IRF8 during induction of the Th9 cell differentiation program(Fig. 3d and Supplementary Fig. 4C). To further study the effect induced by IRF8 deficiency in Th9 cells, we used Enrichr’s web-based tools to perform a comprehensive gene set enrichment analysis, which facilitates the biological interpretation of large data sets and helps to determine functional gene networks^18,19^. We observed that most upregulated and downregulated genes were involved in cytokine or chemokine pathways. In addition, we also observed clusters of genes related to the organisation of extracellular matrix, or fatty acid metabolism (Supplementary Fig. 5). This analysis supported the hypothesis that IRF8 is essential in the regulation of immune functions of Th9 cells.

To determine whether IRF8 directly regulates the Th9 program, we performed IRF8 chromatin-immunoprecipitation (ChIP) sequencing. The IRF8-binding peaks in Th9 cells were located in intronic (29.96%), exonic (1.61%), intergenic (52.15%), promoter (4.05%) and in enhancer (12.23%) regions (Fig. 3e). When we focused on the two main Th9 cytokine genes, *Il9* and *Il21*, which expression were reduced in Th9^IRF8−/−^ (Fig. 3b), we observed a binding of IRF8 to their promoter at −680 and −1048, respectively (Fig. 3f, g). Interestingly, IRF8 interactions on *Il9* and *Il21* genes are located in conserved non-coding sequence (CNS1) of these genes. Using conventional ChIP assay, we confirmed the binding of IRF8 on CNS1 in *Il9* and *Il21* promoters in Th9 but not in Th2 cells (Fig. 3h).

Together these data show that IRF8 transcriptionally activates Th9 differentiation by directly binds to the main Th9-related genes.

### IRF8 needs cooperative factors to induce Th9 polarisation

**Fig. 4:**
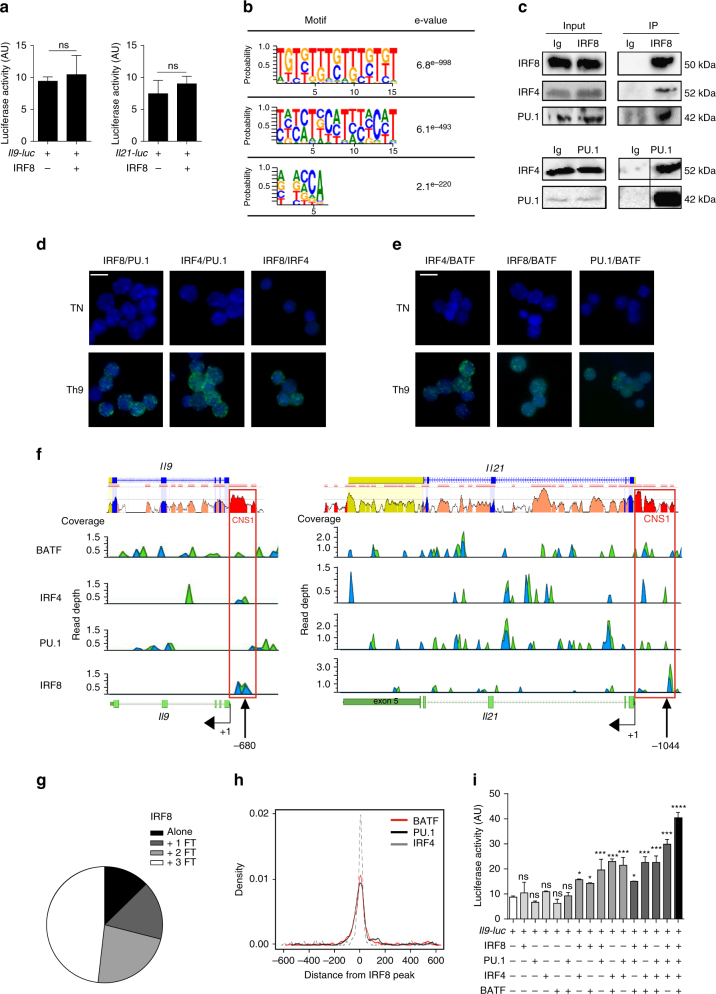
IRF8 needs cooperative factors to induce Th9 polarisation. **a** Transactivation of the *Il9* (left) and *Il21* (right) promoters by IRF8. *Il9* or *Il21* promoter reporter plasmids were transfected into NIH-3T3 cells with an IRF8-encoding vector. **b** Determination of IRF8-binding motifs by analysis of IRF8 ChIP-Seq peaks from WT Th9 cells. Left, letter size indicates frequency of nucleotide. Right, significance of motif occurrence. **c** Immunoprecipitation assay on Th9 cells differentiated for 24 h using anti-IRF8 antibody (above) or anti-PU.1 antibody (below), followed by immunoblot analysis with indicated specific antibodies. Ig is used as a control. **d**, **e** PLA in WT naive CD4^+^ T cells and in Th9 cells after 24 h of differentiation. DAPI staining (blue) indicates the nucleus. Green dots represent proximity between IRF8/PU.1, IRF4/PU.1 or IRF8/IRF4 (**d**) or between BATF and IRF8 or PU.1 or IRF4 (**e**) Magnifications are ×63, scale bar represents 5 µm. **f** Top panel represent a screenshot from the ECR (evolutionary conserved regions) Browser web site of the mouse *Il9* (left) and *Il21* (right) genes. Exons are in blue, introns in pink, UTRs in yellow and CNS are in red. Bottom panel is a representation of binding peaks of ChIP-Seq data of BATF, IRF4, PU.1 and IRF8 in WT Th9 cells differentiated for 24 h. The red square shows the overlap between BATF, IRF4, PU.1 and IRF8 peaks in *Il9* or *Il21* CNS1. **g** Co-location of IRF8, PU.1, IRF4 and BATF-binding peaks in Th9 cells were determined by multiple overlap analysis of the respective ChIP-Seq data. IRF8 can bind DNA alone or in combination with one, two or three factors (IRF4, BATF and PU.1). **h** The average density of IRF4, PU.1 and BATF in Th9 cells is shown for regions centred on the summits of the IRF8 peaks. **i** Transactivation of the *Il9* promoters by IRF8, PU.1, IRF4 and BATF. *Il9* promoter reporter plasmids were transfected into NIH-3T3 cells with vectors encoding IRF8, PU.1, IRF4 and BATF alone or in combination. ns, not significant; *P < 0.05, **P < 0.01; ***P < 0.001 (Kruskal–Wallis test (**a**) or two-way ANOVA (**i**)). Data are from three independent experiments (mean and s.e.m.)

To determine whether IRF8 could directly induce transactivation of Th9-related cytokines, we cloned the *Il9* and *Il21* promoters into a Luciferase Reporter Plasmid. *Il9* or *Il21* reporter plasmids were transfected alone or with an IRF8-expressing plasmid in NIH-3T3 cells. IRF8 alone did not significantly activate the *Il9* and *Il21* promoters, which raised the assumption that a partner is needed (Fig. 4a). To better document the function of IRF8 during Th9 cell differentiation, we used the ChIPMunk tool^20^ and the MEME-ChIP tool^21^ to characterise the IRF8-binding sequence. We identified the 5ʹ-tbtstbtvtktbtbt-3ʹ motif as most often represented in our IRF8 ChIP-Seq experiment (Fig. 4b). We analysed this motif using the Tomtom motif comparison tool (http://meme-suite.org/tools/tomtom), and observed that this sequence matches to the IRF8 consensus-binding sequence (Supplementary Fig. 6A) and interestingly, this motif overlaps PU.1 and IRF4 consensus-binding sequences (Supplementary Fig. 6A). We have therefore hypothesised that IRF8 can interact with PU.1 and/or IRF4 during Th9 cell differentiation. To address this question, we performed immunoprecipitation of IRF8 and analysed whether PU.1 and IRF4 were co-immunoprecipitated. The data indicated that PU.1 and IRF4 can both interact with IRF8 and can also interact with each other (Fig. 4c). To confirm these data, we used Proximity Ligation Assays (PLA) as a functional immunoprecipitation at the cell level and similar interactions between IRF8, IRF4 and PU.1 were observed (Fig. 4d). It has been reported that IRF4 and BATF (basic leucine zipper transcription factor, ATF-like) cooperate in the development of Th9 cells^10^; this is why we wondered whether BATF could also interact with IRF8, PU.1 and IRF4. To address this question, we performed PLAs between BATF and the other transcription factors. Our data confirmed the presence of IRF4/BATF-interacting dots in Th9 cells, but also showed the existence of interactions between IRF8 and BATF and between BATF and PU.1 (Fig. 4e). To confirm these data, we used ChIP-Seq experiments to detect the binding of the four proteins to the *Il9* and *Il21* promoters. Using this strategy, we observed that IRF4, IRF8, PU.1 and BATF bind to the *Il9* and *Il21* promoter on their respective CNS1 (Fig. 4f). The presence of these transcription factors in *Il9* and *Il21* CNS1 was confirmed by conventional ChIP assay (Supplementary Fig. 6B, C). We performed an overall analysis of the binding of IRF8 to the entire Th9 cell genome and observed that approximately half of the IRF8 bindings were detected together with IRF4, PU.1 and BATF (Fig. 4g, h).

To study whether IRF8 in cooperation with IRF4, PU.1 and BATF could induce transactivation of Th9-related cytokines, we used the *Il9* and *Il21* reporter plasmids previously described, transfected into NIH-3T3 cells with IRF8-expressing, PU.1-expressing, IRF4-expressing and BATF-expressing plasmids, alone or in combination. We observed that each transcription factor alone had no significant effect on *Il9* and *Il21* transactivation. Interestingly, co-transfection of IRF8 with one or two additional transcription factors had a slight but significant effect on *Il9* and *Il21* promoter activity. The presence of the four transcription factors was required for maximal activation of *Il9* and *Il21* (Fig. 4i and Supplementary Fig. 6D).

Together these data emphasise that IRF8 is involved in a transcriptional factor complex that includes IRF4/BATF and PU.1. All members are required for complete Th9 differentiation.

### IRF8 is essential for *Il4* repression in Th9 cells

**Fig. 5:**
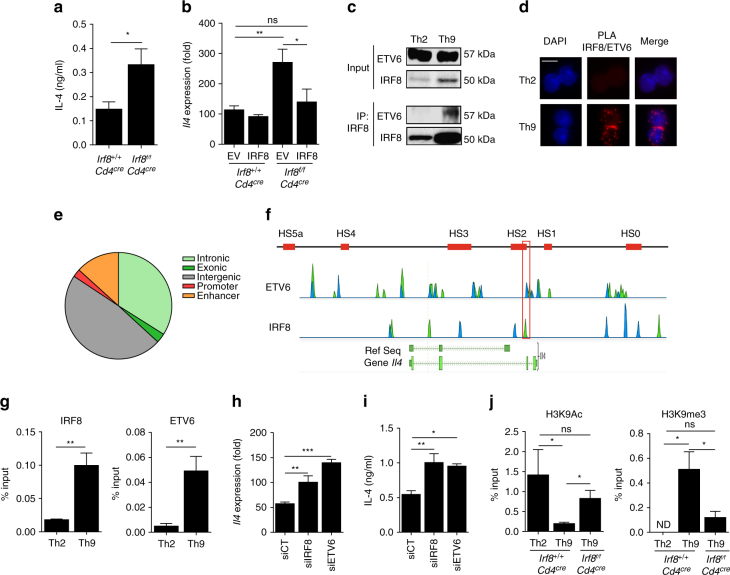
IRF8 is essential for *Il4* repression in Th9 cells by epigenetic mechanisms. **a** *Irf8* ^*+/+*^ *Cd4* ^*cre*^ or *Irf8* ^*f/f*^ *Cd4* ^*cre*^ T cells were differentiated into Th9 for 3 days. IL-4 protein release was assessed by ELISA. **b** WT and *Irf8* ^*f/f*^ *Cd4* ^*cre*^ naive CD4^+^ T cells were retroviral infected with an empty-GFP vector (EV) or IRF8-GFP overexpressing vector (IRF8). GFP^+^ cells were sorted 2 days after infection and differentiated for 3 days into Th9 cells. *Il4* mRNA expression was analysed. **c** Immunoprecipitation (IP) assay using anti-IRF8 antibody on CD4^+^ T cells differentiated into Th2 or Th9 cells for 24 h, followed by immunoblot analysis with indicated specific antibodies. Ig is used as a control. **d** Proximity ligation assay between IRF8 and ETV6 (red dots) in WT Th2 or Th9 cells after 24 h of differentiation. DAPI staining (blue) indicates the nucleus. Magnification are ×63, scale bar represent 5 µm. **e** Distribution of ETV6 ChIP sequencing peaks in WT Th9 cells. **f** Top panel, schematic representation of DNase I hypersensitive sites (red boxes). Bottom panel, Chip-sequencing analysis of the *Il4* gene after Chip-Seq for ETV6 and IRF8 in Th9 cells differentiated for 24 h. The overlap between ETV6 and IRF8 at HS2 locus is framed in red. **g** ChIP assay of IRF8 (left) or ETV6 (right) to the HS2 locus of *Il4* in Th2 and Th9 cells differentiated for 6d. **h**, **i** WT naive CD4^+^ T cells were transfected with siCT or siIRF8 or siRNA against *Etv6* (siETV6), and then polarised under Th9 conditions 3d. Relative expression of *Il4* mRNA (**h**) ELISA analyses of IL-4 protein in supernatant (**i**). **j** ChIP analysis of the binding of H3K9Ac (left) and H3K9me3 (right) at the HS2 locus of *Il4* in Th2, Th9 and Th9^IRF8*−/−*^ cells. ns, not significant; *P < 0.05, **P < 0.01; ***P < 0.001 (Mann–Whitney test (**a**, **g**)) or Kruskal–Wallis test (**b**, **h**–**j**). Data are from four (**g**–**j**) or five (**a**, **b**) independent experiments (mean and s.e.m.)

While deletion of IRF8 decreased the expression of certain Th9 genes (Fig. 1), IRF8 deficiency also increase expression of 217 genes in Th9 cells by more than twofold (Fig. 2a). Interestingly, while *Irf8* downregulation did not induce transdifferentiation of Th9 cells, we observed an increase in Th2-related cytokine production by *Irf8* siRNA (Supplementary Fig. 1B, C). Similar results were observed in Th9^IRF8−/−^, which secreted more IL-4 than their control (Fig. 5a). In addition, IRF8 overexpression in Th9^IRF8−/−^ blunted *Il4* expression (Fig. 5b). These data led us to hypothesise that IRF8 could harbour a repressive transcriptional function in Th9 cells. In macrophages, IRF8 was shown to heterodimerise with ETV6 (ETS variant 6) transcription factor. ETV6 is a ubiquitously expressed member of the Ets (E twenty six) family^22^ (Supplementary Fig. 7A) and is known to exert transcriptional repression through epigenetic modifications^12,23^.

In Th9 cells, we observed using immunoprecipitation and PLA that IRF8 could interact with ETV6 in Th9 cell nuclei (Fig. 5c, d). We observed no dots in Th2 cells (Fig. 5c, d). ETV6 Chip-Sequencing showed 1957 binding sites, 33.95% of which were located in intronic regions, 2.87% in exonic regions, 47.48% in intergenic regions, 2.41% in promoter regions and 13.26% in enhancer regions (Fig. 5e). We found a binding between IRF8 and ETV6 on several genomic sites at the *Il4* locus in Th9 cells (Fig. 5f). In Th2 cells, we did not observe ETV6-binding peaks to the *Il4* locus (Supplementary Fig. 7B). *Il4* gene expression is tightly regulated at the epigenetic level. It has been reported that several loci (CNS 1 and 2, and DNase I hypersensitive sites (HS) HSII to HSVa) may be involved in the regulation of *Il4* expression^24,25^. In Th9 cells, we observed by a ChIP-sequencing analysis that IRF8 and ETV6 bound in close proximity on the HS2 site (Fig. 5f). We confirmed by ChIP assay that IRF8 and ETV6 can interact on HS2 in Th9 cells (Fig. 5g). To validate the hypothesis that ETV6 and IRF8 inhibit *Il4* transcription, we demonstrated that siRNA-targeting IRF8 or ETV6 similarly enhanced *Il4* mRNA expression and IL-4 cytokine secretion in Th9 cells without inducing transdifferentiation (Fig. 5h, i and Supplementary Fig. 7C, D, E).

IRF8 and ETV6 have been shown to interact together to repress gene expression by epigenetic modifications^12,23^. In order to validate the hypothesis that IRF8 could drive epigenetic modifications on HS2 of the *Il4* locus, we carried out ChIP experiments using H3K9me3-specific and H3K9ac-specific antibodies, which target repressive and activating epigenetic marks, respectively. We observed the presence of H3K9me3 repressive mark on HS2 of the *Il4* locus in Th9 cells whereas H3K9ac activating mark was not detected. An opposite observation was observed in Th2 cells (Fig. 5j). Interestingly, in IRF8-deficient Th9 cells, ETV6 failed to bind HS2 of the *Il4* locus (Supplementary Fig. 7F) and we observed a concomitant enrichment of H3K9Ac at this locus (Fig. 5j). Similarly, the enrichment of H3K9me3 repressive mark on HS2 of *Il4* locus was totally blunted in IRF8-deficient Th9 cells (Fig. 5j).

Taken together, these data demonstrate that IRF8 can act as a transcription repressor via ETV6 and induce the H3K9me3 repressive epigenetic mark at HS2 site of *Il4* locus.

### IRF8 is required for the anti-tumour effect of Th9 cells

**Fig. 6:**
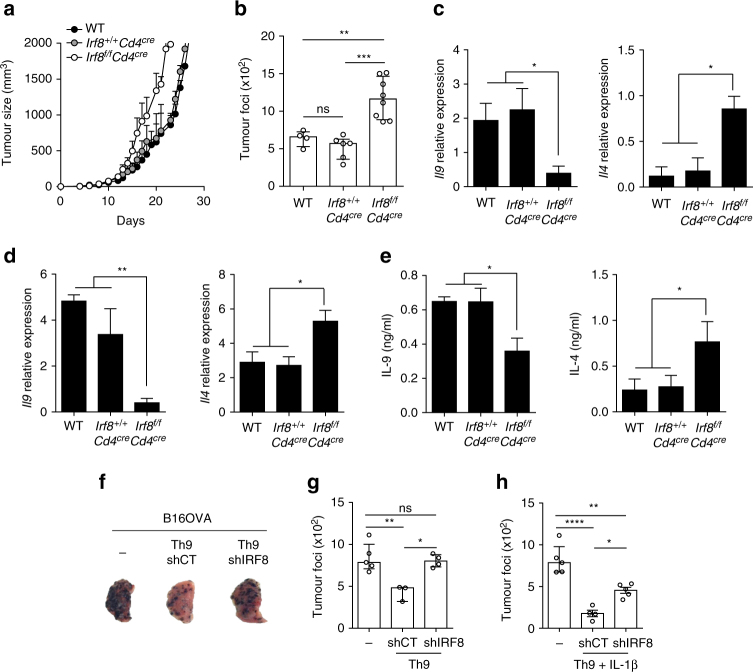
IRF8 is required for anti-tumour effect of Th9 cells. **a** WT, *Irf8* ^*f/f*^ *Cd4* ^*cre*^ and *Irf8* ^*+/+*^ *Cd4* ^*cre*^ mice were subcutaneously (s.c.) injected with B16F10 cells and tumour growth was monitored. Representative data from one of three experiments are shown (mean and s.e.m., 10 mice per group). **b** WT, *Irf8* ^*f/f*^ *Cd4* ^*cre*^ and *Irf8* ^*+/+*^ *Cd4* ^*cre*^ mice were intravenously (i.v.) injected with B16F10 melanoma cells and 12 days later mean numbers of lung tumour foci are shown (mean and s.e.m., 5 mice per group, 3 independent experiments). **c** *Il4* and *Il9* mRNA expression analysis in tumour infiltrated lymphocytes (TILs). **d**, **e** Tumour-draining lymph nodes (TDLN) from WT, *Irf8* ^*f/f*^ *Cd4* ^*cre*^ and *Irf8* ^*+/+*^ *Cd4* ^*cre*^ mice given a subcutaneous injection of B16F10 cells were collected at day 13. Analysis of *Il4* and *Il9* mRNA expression in TDLN (**d**). TDLN cells were stimulated for 72 h in plate-bound anti-CD3 and anti-CD28, and IL-4 and IL-9 release was assessed by ELISA (**e**). **f**–**h** WT mice were injected iv with B16F-OVA melanoma cells or together with effector OT-II Th9 (**f**, **g**) or OT-II Th9 + IL-1β (**h**) cells infected with shRNA control (shCT) or against *Irf8* (shIRF8). Representative lung from mice untreated or treated with Th9 cells infected with shCT or shIRF8, 13 days after intravenous injection (**f**). Mean numbers of lung tumour foci are shown (**g**, **h**) (mean and s.e.m., 5 mice per group, 3 independent experiments) ns, not significant; *P < 0.05, **P < 0.01; ***P < 0.001 (Kruskal–Wallis test (**b**–**e**, **g**, **h**))

Th9 cells are potent anti-tumour cells in the context of melanoma. To determine the effects of IRF8 in Th9 cell development during melanoma growth, we used the B16F10 model and studied tumour growth in WT, *Irf8* ^*f/f*^ *Cd4* ^*cre*^ and *Irf8* ^*+/+*^ *Cd4* ^*cre*^ mice. We observed that IRF8 deficiency in CD4^+^ T cells favoured melanoma growth in both lung metastasis and subcutaneous tumour models (Fig. 6a, b). In these models, we observed that *Il9* mRNA expression and IL-9 protein secretion were reduced in tumour-infiltrating CD4^+^ T cells (TILs) or in CD4^+^ T cells from tumour-draining lymph nodes (TDLN) of *Irf8* ^*f/f*^ *Cd4* ^*cre*^ mice compared to those of WT or *Irf8* ^*+/+*^ *Cd4* ^*cre*^ mice (Fig. 6c–e). Interestingly, we also observed higher *Il4* expression at the mRNA and protein levels in CD4^+^ T cells from *Irf8* ^*f/f*^ *Cd4* ^*cre*^ compared to WT and *Irf8* ^*+/+*^ *Cd4* ^*cre*^ mice (Fig. 6c–e). When we analysed using RTqPCR the expression of key transcription factors and cytokines specific to the other CD4 T-helper subtypes, we found no accumulation of other particular helper T cells in *Irf8* ^*f/f*^ *Cd4* ^*cre*^ compared with WT and *Irf8* ^*+/+*^ *Cd4* ^*cre*^ mice (Supplementary Fig. 8A). We monitored the expression of certain genes that were found to be either upregulated or downregulated by IRF8 in our in vitro experiments (Fig. 3b) and observed that IRF8 deficiency altered the CD4^+^ T-cell transcriptional program in a similar way to that found in vitro (Supplementary Fig. 8B). To more directly determine the ability of Th9 cells to control melanoma growth, we used the adoptive transfer of tumour-specific Th9 cells, as we previously published that adoptive transfer of Th9 cells had an anti-tumour effect^8^. We isolated naive CD4^+^ T cells from OT-II mice. These cells were retrovirally infected with a sh-control-GFP or a sh-*Irf8*-GPF vector and then differentiated into Th9. Importantly; *Irf8* downregulation dramatically decreased *Il9* and *Il21* expression, and enhanced *Il4* expression without affecting *Ifnγ* and *Il17a* or master controller transcription factor expressions (Supplementary Fig. 8C and D). We then intravenously transferred differentiated T-helper cells into syngeneic immunocompetent WT mice concomitantly inoculated with melanoma cells expressing full-length ovalbumin (B16-OVA). We observed that sh-control-infected Th9 OT-II cells exerted an anti-tumour effect on the lung foci of B16-OVA tumours. In contrast, Th9 OT-II cells infected with *Irf8* shRNA had a reduced anti-tumour effect (Fig. 6f, g). We have recently reported that Th9 cells have improved anti-tumour properties when differentiated in the presence of IL-1β. To determinate whether IRF8 could impair the anti-tumour functions of Th9 cells differentiated with IL-1β, we used the same strategy. Similarly, IRF8 deficiency in these cells results in a decrease in *Il9* and *Il21* expressions in favour of an increase in *Il4* expression (Supplementary Fig. 8C, D). Sh-control-Th9 cells differentiated in the presence of IL-1β harboured a strong anti-tumour effect, which was completely lost when cells were infected with Sh-*Irf8* (Fig. 6h). These data emphasise the major involvement of IRF8 in Th9 cells anti-tumour functions.

## Discussion

In this report we unravelled the essential function of IRF8 during T-helper (Th)9 polarisation in vitro and in vivo. We hypothesised that IRF8 had two transcriptional functions in Th9 cells, and that these depended on its partners. First, IRF8 interacts with PU.1, IRF4 and BATF (basic leucine zipper transcription factor, ATF-like) and this large molecular transcriptional complex is essential for the induction of Th9 polarisation and the induction of Th9 cell-related cytokines, such as IL-9 and IL-21. Second, IRF8 can interact with ETV6 (Ets variant 6) to form a repressive transcriptional complex that acts through epigenetic modulations and notably blunts the ability of Th9 cells to produce Th2 cell-specific cytokines, such as IL-4, by increasing repressive marks.

IRF8 is one of the nine members of the IRF family. It shares strong homologies with IRF4. It is expressed by B cells, DCs, macrophages^26–28^ and activated T cells^29,30^. This protein has been shown to support various functions in the regulation of both innate and adaptive immune responses. According to its partner, IRF8 has been shown to bind to various DNA sequences and thus act either as an activator or a repressor^31–33^. IRF8 can form heterodimers with Ets transcription factors such as PU.1 to activate transcription, driven by the Ets–IRF (EICE) or the IRF–Ets composite sequence (IECS). IRF8 also interacts with AP-1 factors, such as BATF3 in DCs, to form the IRF8–BATF3–JUN complex binding to AP-1–IRF composite sequence (AICE) to promote gene activation^34^. The association of IRF factors with AP-1 or Ets factors was thought to be exclusive. However, our report shows that complexes involving IRF8 associated with either AP-1 or Ets factor could occur in the same cell.

IRF4 and IRF8 have similar molecular structures and both recognise the same DNA sequences. It was therefore assumed that both factors were likely to function redundantly to regulate expression of the same target genes^35^. In B cells, PU.1 and IRF8 are expressed during early B-cell differentiation and IRF4 mainly at a later stage. Concomitant expression of PU.1, IRF4 and IRF8 is only observed in activated B cells^36^. Contrast patterns of IRF8 and IRF4 expression and functions suggest that in B cells, these two factors could have competitive and antagonistic functions by binding to the same regulatory regions but resulting in opposite transcriptional effects^36^. Our report adds another level of complexity in the function of IRF4 and IRF8 showing the first evidence of their direct interaction, suggesting that these factors are not only redundant or competing molecules, but could also be cooperative partners. In addition, IRF8 could interact in the same complex with IRF4 and also with BATF and PU.1 resulting in the formation of a large transcriptional complex responsible for transcriptional activation of Th9 cell-specific cytokines, *Il9* and *Il21*. It remains to be determined whether other transcription factors or epigenetic modulators such as STATs (signal transducer and activator of transcription) or histone acetyl transferases or demethylases may also participate in this complex to modulate Th9 cell-specific cytokine expression.

Many studies have demonstrated that IRF8 has critical functions in the differentiation of myeloid cells, favouring monocyte cells rather than granulocyte cells differentiation^37^. It is also required for DC differentiation and functions^38,39^. In lymphoid cells, IRF8 is involved in B-cell lineage specification, immunoglobulin light chain gene rearrangement, distribution of mature B cells in the marginal zone and follicular B-cell compartment, and transcriptional regulation of critical elements of the germinal centre reaction, such as BCL6 (B-cell lymphoma 6) and activation-induced cytosine deaminase^40^. In the context of CD4^+^ T cells, IRF8 is not expressed in naive T cells, but is rapidly induced in response to antigenic stimulation or TCR activation^41^, suggesting that it may be necessary to regulate genes involved in T-cell differentiation and/or their effector functions. A previous study described IRF8 as being involved in Th17 cell differentiation^13^, then Kim et al. confirmed it by observing an increased production of IL-17 in activated CD4^+^ T cells derived from *Irf8* ^*f/f*^ *Cd4* ^*cre*^ mice^17^. Surprisingly, IRF8 function during Th17 differentiation was not related to its transcriptional function. IRF8 has been shown to inhibit Th17 cell differentiation by its physical interaction with RORγt, leading to RORγt degradation^13^. However, another group failed to confirm the involvement of IRF8 in Th17 cell differentiation^42^. IRF8 also appears to be involved in Treg cell development and functions. Lack of IRF8 in these cells reduced their ability to migrate to an inflammatory site. Interestingly, these Treg cells also exhibit aberrant expression of Th2- and Th17-cell-related cytokines^15^. However, the molecular mechanisms underlying this observation are not explained. Therefore, while it seems clear that IRF8 is expressed in many CD4^+^ T-cell subsets, its function is unclear and especially its transcriptional function. Our data provide the first evidence that IRF8 could control the transcriptional program of a Th subset.

PU.1, a transcription factor of the Ets family, is a key transcription factor in Th9 differentiation. *Il9* transcription is controlled by PU.1, which binds directly to *Il9* promoter, where it forms a complex with histone acetyltransferase GCN5. GCN5 induces epigenetic modifications at the *Il9* locus and brings about the *Il9* promoter activity^43^. Therefore, ectopic expression of PU.1 enhances IL-9 production, whereas PU.1 deficiency in T cells results in markedly decreased IL-9 production^4^. Different partners of PU.1 have been described^44^ and IRF4 and IRF8 have been reported to heterodimerise with PU.1 in B cells and myeloid cells^45,46^. Our work demonstrated that these three proteins interact together with BATF to induce transcription of Th9 cytokines. IFR4 was reported to heterodimerise with BATF in Th17 and in Th9 cells^10,47^, but the identity of the PU.1 partner remained unknown. Our report provides a clearer view of the organisation of the molecular complex which drives Th9-related cytokine transcription.

Another important point is the ability of IRF8 to regulate IL-4 production. In Th9 cells, IL-4 induces STAT6 activation and GATA3 expression that are classically involved in *Il4* transcription. A previous report suggests that Foxp3 and GATA3 may physically interact with each other, thus limiting the ability of GATA3 to induce *Il4* expression. However, Foxp3 expression in Th9 cells remains modest^1^. The SMAD pathway has also been reported to downregulate the expression of Th2 cell-related cytokines^48,49^. PU.1 could also limit the production of Th2 cell-related cytokines at least in part by limiting the ability of GATA3 and IRF4 to interact with target loci that encode cytokines^9^. We propose here that IRF8 could be another regulator of Th2 cell-related cytokines in Th9 cells. Indeed, the IRF8/ETV6 heterodimer could regulate the expression of *Il4* in Th9 cells by epigenetic modifications of the *Il4* locus. The IRF8/ETV6 heterodimer has been described in macrophages. ETV6 induces the recruitment of the HDAC3 (histone deacetylase 3) histone deacetylase thus leading to transcriptional repression^12^. *Il4* expression is tightly regulated at epigenetic levels. The *Il4* locus contains cis-regulatory elements such as CNS or HS harbouring enhancer or silencer functions. Our study indicates that the IRF8/ETV6 complex can bind to HS2 on the *Il4* locus, leading to epigenetic modifications such as H3K9 deacetylation and trimethylation in Th9 cells. In IRF8-deficient Th9 cells we found no interaction of ETV6 with the *Il4* locus, and therefore H3K9 was not methylated but acetylated at the *Il4* locus. In the absence of IRF8, Th9 cells expressed *Il4*. We therefore hypothesise that the IRF8/ETV6 complex is a major actor involved in *Il4* repression during Th9 differentiation.

Here we show that IRF8 deficiency does not only dampen IL-9 production from Th9 cells, but also enhances IL-4 secretion by Th9 cells. These two cytokines are critical for asthma development, and it might be interesting to know if IRF8 deficiency in Th9 cells has an impact on this atopic disease.

To sum up, our work unravels the essential involvement of IRF8 in the control of the Th9 cell transcriptional program. It assumes a dual function that depends on its partner and leads to *Il9* and *Il21* transcription and *Il4* repression. These results raise the question of testing the function of IRF8 in other T-helper subsets and suggest that a global view of its partners is necessary to understand its function in a particular cellular context. In vivo, IRF8 is essential for Th9 cell generation and their biological functions in the context of cancer. This result emphasises that activation of the transcriptional function of IRF8 could be a therapeutic tool to stimulate or inhibit Th9 cell functions and could be useful in a context such as cancer.

## Methods

### Mice

All animals were maintained in specific pathogen free facility at Dijon Zootechnical Centre. Animal use and care were approved by the Burgundy University Animal Experiments Ethics Committee (approved protocol #2212), in accordance with the Federation of Laboratory Animal Science Associations (FELASA). Only females between 6 and 10 weeks of age were used for the experiments. Female C57BL/6 J were purchased from Charles River Laboratories (Saint-Germain sur l’Arbresle, France). IL-9-eGFP mice were provided by R. Flavell. OT-II mice and *Irf8* ^*f/f*^ *Cd4* ^*cre*^ mice were provided by the CDTA (Cryopréservation, Distribution, Typage et Archivage animal) and distributed by EMMA (European Mouse Mutant Archive, a service funded by the EC FP7 Capacities Specific Programme).

### In vitro T-cell differentiation

Naive CD4^+^ T cells (CD4^+^ CD62L^+^) were obtained from spleens and lymph nodes of C57BL/6 WT or *Irf8* ^*f/f*^ *Cd4* ^*cre*^ mice. CD4^+^ T cells were purified from spleen and lymph nodes with anti-CD4 microbeads (130-093-227, Miltenyi Biotec), then were further sorted as naive CD4^+^CD62L^+^ T cells. The purity of the isolated naive T-cell population routinely exceeded 95%. Naive T cells were cultured for three days with anti-CD3 (2 µg/ml) and anti-CD28 (2 µg/ml) antibodies in the presence of anti-IFNγ (10 µg/ml) and anti-IL-4 (10 µg/ml) to obtain Th0, anti-IL-4 (10 µg/ml) and IL-12 (10 ng/ml) to obtain Th1, anti-IFNγ (10 µg/ml) and IL-4 (10 ng/ml) to obtain Th2, anti-IFNγ (10 µg/ml), IL-4 (20 ng/ml) and TGF-β (2 ng/ml) to obtain Th9, anti-IFNγ (10 µg/ml), anti-IL-4 (10 µg/ml), IL-6 (20 ng/ml) and TGF-β (2 ng/ml) to obtain Th17 and anti-IFNγ (10 µg/ml), anti-IL-4 (10 µg/ml) and TGF-β (4 ng/ml) to obtain Treg. Cells were cultured at 37 °C under 5% CO_2_ in RPMI 1640 with 10% (vol/vol) fetal calf serum supplemented with sodium pyruvate, penicillin and streptomycin, and 4 mM of 4-(2-hydroxyethyl)-1-piperazineethanesulfonic acid (HEPES). Anti-CD3 (Armenian Hamster IgG, clone 145-2C11, #BE0001-1), anti-CD28 (Armenian Hamster IgG, clone PV-1, #BE0015-5), anti-IL-4 (Rat IgG1, clone 11B11, #BE0045) and anti-IFN-γ (Rat IgG1, clone XMG1.2, #BE0055) antibodies were obtained from BioXcell (West Lebanon, NH, USA) and IL-12, IL-6, TGF-β and IL-4 were purchased from R&D Systems.

For in vitro treatments, SMAD3 inhibitor (SIS3, 10 µM, 1 h), TGF-β receptor kinase inhibitor (SB431542, 5 µM, 1 h), p38 inhibitor (SB203580, 10 µM, 1 h), JNK inhibitor (SP600125, 10 µM, 1 h) and the ROCK inhibitor (Y27632, 10 µM, 1 h) were obtained from Merck Chemical France.

### Reverse transcription PCR and quantitative PCR analysis

Total RNA from T cells was extracted using Trizol (Invitrogen, Carlsbad, CA, USA). In total, 300 ng of RNA were reverse-transcribed into cDNA using M-MLV reverse transcriptase (28025-013, Invitrogen), Random Primers and RNAseOUT inhibitor (10777-019, Invitrogen). cDNA were quantified by real time PCR using a Power SYBR Green Real-time PCR kit (4367659, Life technologies) on a 7500Fast detection system (Life technologies). Relative mRNA levels were determined using the ∆Ct method. Values were expressed relative to β-actin unless otherwise specified. The sequences of the oligonucleotides used are described in Supplementary Table 1.

### ELISA

After polarisation for 72 h, cell culture supernatants were assayed by ELISA for mouse IL-9 (442704, BioLegend), IL-4 (555232, BD Biosciences), IL-5 (555236, BD Biosciences), IL-13 (88-7137-88, eBiosciences), IL-17A (432501, Biolegend) and IFNγ (555138, BD Biosciences) according to the manufacturer’s protocol.

### Western blotting and immunoprecipitation assays

Cells were lysed in boiling buffer (1% SDS, 1 mM sodium orthovanadate and 10 mM Tris (pH 7.4)) containing protease inhibitor cocktail for 20 min at 4 °C. Cell lysates were subjected to sonication (10 s at 10%) and protein concentration was assessed using the Bio-Rad DC Protein Assay Kit (5000112, Bio-Rad). Proteins were then denaturated, loaded and separated on SDS-PAGE and transferred on nitrocellulose membranes (Schleicher & Schuell). After blocking with 5% non-fat milk in phosphate-buffered saline containing 0.1% Tween 20 (PBST), membranes were incubated overnight with primary antibody (Supplementary Table 2) diluted (1 μg/ml) in PBST containing 5% BSA, washed and incubated for 1 h with secondary antibody diluted in PBST-5% non-fat milk. After additional washes, membranes were incubated with luminol reagent (Santa Cruz Biotechnology). Uncropped full-length blots of data in this article are presented in Supplementary Fig. 9.

Immunoprecipitation assays were done with at least 50 × 10^6^ Th9 cells. Briefly, cells were lysed in 1 ml lysis buffer (20 mM Tris (pH 7.5), 15 mM KCl, 1% CHAPS, 5 mM MgCl_2_, 1 mM EDTA, 1 mM EGTA and CPIM) for 30 min on ice. After centrifugation at 14,000 × *g* at 4 °C for 30 min, supernatants were precleared for 2 h at 4 °C in the presence of 30 μl of mixed Sepharose 6B (6B100, Sigma-Aldrich) and protein G (17-0618-01, Amersham, GE Healthcare, Velizy-Villacoublay, France). After centrifugation at 1000 × *g* for 3 min the supernatant was incubated with anti-IRF8 antibody (Supplementary Table 2) or rabbit-negative control immunoglobulin (Supplementary Table 2) (5 μg/ml) and with 40 μl of mixed Sepharose overnight at 4 °C. The precipitates were washed four times in lysis buffer and analysed by immunoblotting.

### ChIP

ChIP was conducted according to the manufacturer’s instructions (53009, ChiP-IT Express Enzymatic; Active Motif). Briefly, 10 × 10^6^ cells were fixed in a solution containing 37% formaldehyde for 10 min and quenched with 0.125 mol/l glycine. Chromatin was isolated and sheared to an average length of 300–500 bp using Enzymatic Cocktail. A total of 7 μg of DNA were immunoprecipitated with 3 μg of pSmad3, IRF8, PU.1, BATF or IRF4 antibodies, or 3 μg of negative control immunoglobulin at 4 °C overnight. After chromatin elution, cross-links were reversed and samples were analysed by quantitative PCR. The sequences of the oligonucleotides used are described in Supplementary Table 1.

### siRNA transfection

siRNA knockdown experiments were performed with validated control, or *Irf8* (s68003), or *Smad3* (s69494) or *Etv6* (s65718)-specific siRNAs (Life Technologies). In brief, naive CD4^+^ T cells were transfected with Transit-TKO transfection Reagent (MIR2154, Mirus) according to the manufacturer’s instructions for 24 h and then differentiated as previously described.

### Promoter-activity reporter assay

The *Il9-luc* and *Irf8-luc* luciferase constructs were obtained by inserting 2390 pb of *Il9* and 1634 pb of *Irf8* mouse promoters in the multicloning site of the pGl3 basic vector (Promega, Charboniere, France). Fragments were amplified by high-fidelity PCR with C57BL/6 mice DNA as the template and specific primers (Supplementary Table 1).

Mouse NIH-3T3 cells (cultured in DMEM 4.5 g/l + glutamine + FBS at 37 °C, 5% CO_2_) were transiently transfected for 48 h with reporter plasmid and the pCMV-SPORT6-IRF8 (GE Healthcare), pCMV-SPORT6-PU.1 (GE Healthcare), pCR4-TOPO-IRF4 (Thermo Scientific) and pcDNA3.1-mBATF (Addgene) using Lipofectamine 2000 (Invitrogen). Luciferase was measured using the Dual Glo Luciferase Assay System (E2920, Promega) according to the manufacturer’s instructions. Briefly, Dual Glo Luciferase Reagent was added to the cells. After 10 min incubation, firefly luciferase was measured with a Wallac 1440 Victor2 luminometer (PerkinElmer, Courtaboeuf, France). The reaction was stopped with Dual-Glo Stop and Glo Reagent for 10 min and the renilla luciferase was then measured.

### In situ proximity ligation assay

In total, 1 × 10^6^ purified naive T cells were differentiated for 24 h into murine Th9 cells. Cells were washed, then fixed for 10 min at 4 °C with 4% PFA and were permeabilised with glacial methanol on ice. Non-specific binding on slides was blocked by incubation for 20 min at room temperature with a buffer of 0.5% BSA in PBS. Samples were then incubated overnight at 4 °C with primary antibodies (Supplementary Table 2). Primary antibodies were washed out, cells were incubated for 1 h at 37 °C with the appropriate probes (anti-Rabbit PLUS, #DUO92002; anti-Goat MINUS, #DUO92006 and anti-Mouse MINUS, #DUO92004) and were washed twice with PBS. Probes were then ligated for 30 min at 37 °C and washed twice in Buffer A and were amplified for 100 min at 37 °C in the dark with polymerase (DUO92008 or DUO92014, Sigma-Aldrich). Cells were then mounted for 2 h in the dark on a drop of Mounting Medium containing DAPI (P36931; Molecular Probes) on a microscopy slide (045796; Dutscher). Slides were imaged with a charge-coupled device-equipped upright microscope (Zeiss) and ×40 or ×63 objectives with a numerical aperture of 1.4. Images were analysed by ImageJ software.

### Retroviral transduction

For retrovirus infection, *Irf8* was cloned into pMYs-IRES-GFP retroviral vector (Cell Biolabs) as previously described^8^. cDNA was amplified by PCR using the following pairs of oligo-nucleotide primers 5′-aactcgagaacaccatgtgtgaccg-3′ and 5′-tagtggcagattatcgccggcgat-3′. The ligation of DNA fragments was performed with T4 DNA ligase (M1801, Promega). The orientation of the insert was determined by PCR and restriction enzyme digestion.

ShRNA specific for *Irf8* (5′-ccaggctttccgcatgtttttcaagagaaaacatgcggaaagcctgg-3′) was cloned into pMXs-U6-GFP retroviral vector (Cell Biolabs). BamHI and EcorI restrictions enzyme sites were introduced for subcloning. The ligation of DNA fragments was performed with T4 DNA ligase (M1801, Promega).

Retroviral particles were generated by transfecting the platinum-E cells with Lipofectamine 2000 (Invitrogen) according to the manufacturer’s instructions. After 2 days, fresh virus supernatant was harvested and mixed with proliferative CD4^+^ naive T cells and 10 μg/ml protamine sulphate (APP Pharmaceuticals) in a 24-well plate and centrifuged for 90 min at 2,000 × *g* at 32 °C. The transduced naive CD4^+^ T cells were collected after 2 days and cell-sorted according to GFP expression. GFP^+^ cells were differentiated as described above. After 3 days, the cells were collected for PCR and ELISA assays.

### Flow cytometry

Cells were stained with different antibodies (Supplementary Table 3) for 15 min at room temperature. After surface staining, 2 ml of red blood cell lysis solution (349202, BD Biosciences) were added for 10 min, centrifuged (400 × *g*, 5 min) and then resuspended in flow cytometry buffer (00-4222-26, eBiosciences). All events were acquired by a BD LSR-II cytometer equipped with BD FACSDiva software (BD Biosciences) and data were analysed using FlowJo software (Tree Star, Ashland, Oregon).

For intracellular staining, cells were cultured for 5 days and then stimulated for 4 h at 37 °C in culture medium containing phorbol 12-myristate 13-acetate (PMA; 50 ng/ml; Sigma-Aldrich), ionomycin (1 μg/ml; Sigma-Aldrich) and monensin (GolgiStop; 1 μl/ml; BD Biosciences). After staining for surface markers, cells were fixed and permeabilised according to the manufacturer’s instructions (00-5521-00, Foxp3 Fixation/Permeabilization kit, eBiosciences), then stained for intracellular IL-9 (APC, RM9A4, Biolegend). FACS-gating strategies are presented in Supplementary Fig. 10.

### NGS analysis

For RNA-Seq library preparation, total RNA from T cells was extracted using Trizol (Invitrogen, Carlsbad, CA, USA). rRNA were removed using the Ribo-zero rRNA Removal Kit (Illumina, San Diego, CA, USA). A total of 100 ng of rRNA-depleted RNA was used for the library preparation using the TruSeq Stranded Total RNA Library Prep kit (Illumina) following the manufacturer’s instructions. RNA sequencing was performed on NextSeq device (Illumina). The RNA-seq libraries were sequenced with paired-end 75 bp reads. FASTQ files were mapped by using BWA (mm10 version of Mus Musculus genome) for Illumina^50^. The analysis was performed by using TopHat for Illumina^51^. Generated files were processed with Cufflinks software^52^ to obtain annotated expressed genes in each studied subtype. Then, differential expression between the samples was analysed with Cuffdiff^52^. Unsupervised hierarchical clustering of samples was performed by using Gene Cluster 3.0 software and viewed with Treeview viewer. Genes were normalised and mean centred. The hierarchical clustering was performed using Correlation measure and complete linkage analysis. Upregulated genes appear in red and downregulated genes in green.

Before the ChIP-Seq experiments, Chromatin from 2 × 10^7^ Th9 cells was prepared by using the Truchip Chromatin Shearing Reagent Kit (Covaris) by following the manufacturer’s instructions. Chromatin shearing was performed with 3 cycles of 12 min of sonication (M-Serie Protocol indicated in the Truchip protocol) with a Covaris S220 (Covaris). ChIP experiments were performed as previously described but, after chromatin elution, 500 ng of purified DNA was used for the library preparation using the NEBNext Ultra DNA library kit for Illumina (New England Biolabs, USA) following the manufacturer’s instructions. DNA sequencing was performed on NextSeq device (Illumina). The DNA-seq libraries were sequenced with paired-end 75 bp reads. Sequenced reads were aligned to the mouse genome (mm10 assembly) with Bowtie 3. The output of Bowtie was converted to BED files using MACS (Version 1.0.1). The Nebula-web^54^ server was then used for peak calling with FindPeaks, de novo motif discovery with ChIPMunk^53^ and statistical analysis. The Golden Helix genome browser software allowed us to visualise the peaks on the mouse genome mm9. Finally, the intersection of RNA-Seq and ChIP-Seq data was illustrated using http://bioinformatics.psb.ugent.be/webtools/Venn/ web site. All peak overlap analyses were performed with Rscripts using a minimal overlap length of one and allowing for all possible overlaps. Results were parsed and converted to tables with custom-made bash and R scripts.

### Tumour growth experiments

B16-F10 and B16-OVA murine melanoma cells were cultured at 37 °C under 5% CO2 in DMEM high glucose with GlutaMax-1 (Lonza) supplemented with 10% (v/v) foetal calf serum (Lonza), 1% penicillin, streptomycin, amphotericin B (Gibco), 4 mmol/l HEPES (Gibco) and 1 mmol/l sodium pyruvate (Gibco). B16F10 cells (ATCC CRL-6475) were obtained from American Type Culture Collection. B16-OVA cells were provided by J.D. Rosenblatt. All cells were routinely tested for Mycoplasma contamination using Mycoalert Mycoplasma Detection Kit (Lonza) and were found to be negative. To induce tumour formation, 2 × 10^5^ B16-F10 or B16-OVA cancer cells were injected subcutaneously into mice. After one week, tumour size were measured daily with an electronic calliper. According to our institutional ethical board, animals were killed by cervical dislocation, after have been anesthetised, when the maximum tumour size (2000 mm^3^) was achieved or when a necrosis superior to 2 mm was observed. Alternatively, 2 × 10^5^ B16-F10 or B16-OVA cells were injected intravenously into mice. Lung tumour foci were counted after 13 days by researchers ‘blinded’ to sample identity. All tumour growth experiments were approved by the Burgundy University Animal Experiments Ethics Committee (approved protocol #2212).

### Statistical analyses

Results are shown as mean ± s.e.m., and data sets was compared using unpaired Student’s *t*-test (test group versus control group) or 2-way ANOVA when appropriate. Differences in tumour foci numbers were assessed using Student’s *t*-test or Kruskall–Wallis test according to group numbers. We performed statistical calculations with GraphPad Prism 5 (La Jolla, CA, USA). All *P* values were two-tailed. A *P* < 0.05 was considered statistically significant for all experiments.

### Data availability

Sequence data that support the findings of this study have been deposited in GEO with the primary accession code GSE99167. The authors declare that all other data supporting the findings of this study are available within the article and its Supplementary Information Files, or are available upon reasonable requests to the authors.

## Electronic supplementary material

Supplementary information
