# Supplementary material for: IRF8-dependent molecular complexes control the Th9 transcriptional program
Source: Nat Commun. 2017 Dec 12;8:2085. doi: 10.1038/s41467-017-01070-w (PMC5727025; doi:10.1038/s41467-017-01070-w)
Supplement: Supplementary file 1 — Supplementary information [file 41467_2017_1070_MOESM1_ESM.pdf]

Supplementary Figure 1

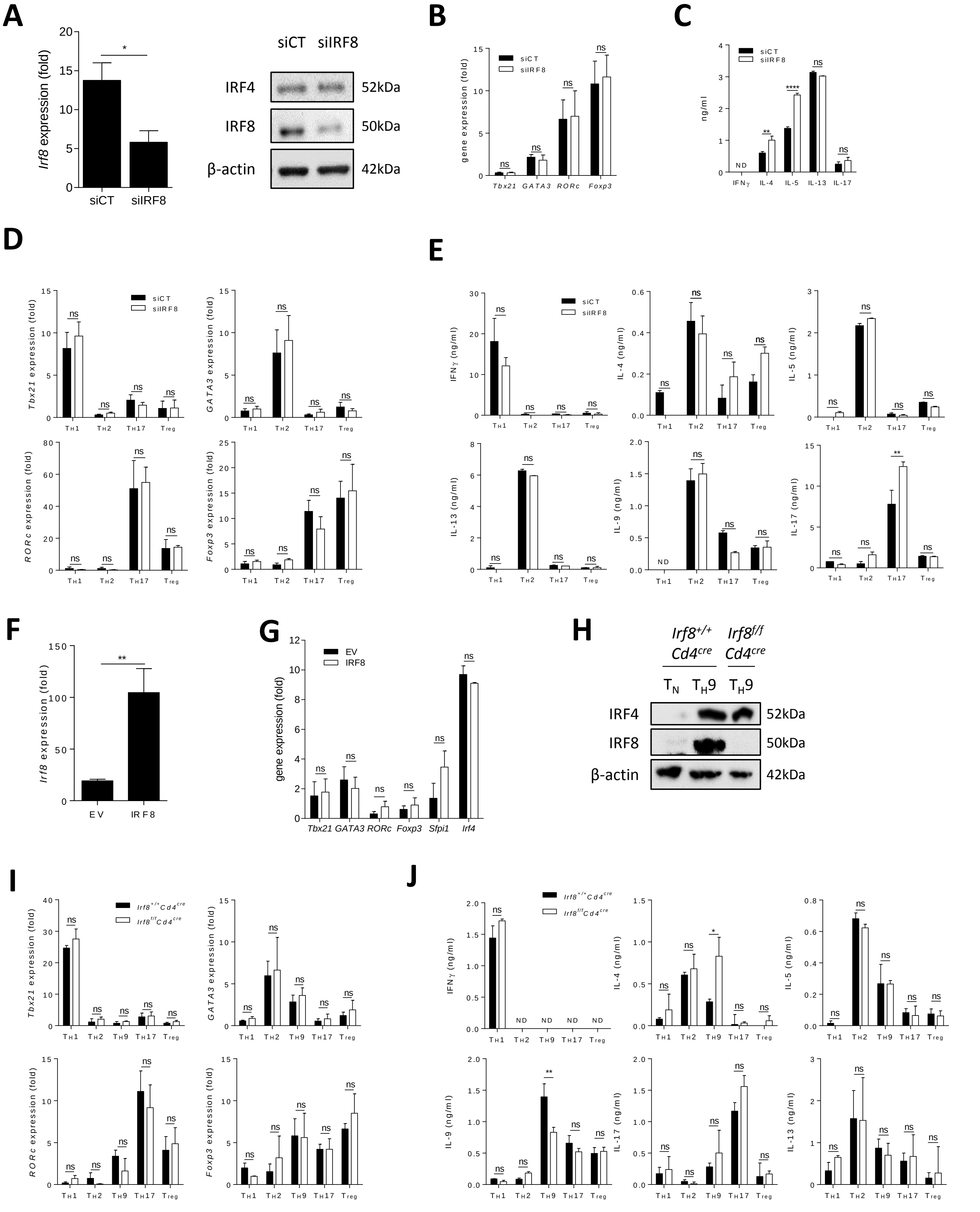

Supplementary Fig.1

A. Left panel, RT-PCR analysis of *Irf8* expression in Th9 cells transfected with siRNA sontrol (siCT) or against IRF8 (siIRF8). Right panel, immunoblot analysis of IRF8 and IRF4 expression in Th9 cells transfected with siCT or siIRF8.

B. Expression of master regulator genes (*Tbx21*, *GATA3*,*RORc* and *Foxp3*) in Th9 cells transfected with siCT or siIRF8.

C. Cytokine release (IFNγ, IL-4, IL-5, IL13 and IL-17) by Th9 cells transfected with siCT or siIRF8.

D. Expression of master regulator genes (*Tbx21*, *GATA3*,*RORc* and *Foxp3*) in Th1, Th2, Th17 and Treg cells transfected with siCT or siIRF8.

E. Cytokine release (IFNγ, IL-4, IL-5, IL13, IL-9 and IL-17) by Th1, Th2, Th17 and Treg cells transfected with siCT or siIRF8.

F. *Irf8* mRNA expression from GFP^{+} cells sorted from naive $CD4^{+}$ T cells 2 days after retroviral infection with empty vector expressing GFP alone (EV) or GFP plus overexpression of IRF8 (IRF8) and differentiated for 1 day into Th9 cells.

G. Expression of master regulator genes (*Tbx21*, *GATA3*,*RORc* and *Foxp3*) and Th9 cells-specific gene (*Sfpi1*, *Irf4*) in Th9 cells infected with EV or IRF8.

H. Immunoblot analysis of IRF8 and IRF4 in naive T $CD4^{+}$ cells (TN) and Th9 cells differentiated from $Irf8^{+/+}Cd4^{cre}$ or $Irf8^{f/f}Cd4^{cre}$ mice.

I. Expression of master regulator genes (*Tbx21*, *GATA3*,*RORc* and *Foxp3*) in Th1, Th2, Th9, Th17 and Treg cells differentiated from $Irf8^{+/+}Cd4^{cre}$ or $Irf8^{f/f}Cd4^{cre}$ mice.

J. Cytokine release (IFNγ, IL-4, IL-5, IL13, IL-9 and IL-17) by Th1, Th2, Th9, Th17 and Treg cells differentiated from $Irf8^{+/+}Cd4^{cre}$ or $Irf8^{f/f}Cd4^{cre}$ mice.

ns, not significant; * $P<0.05$, ** $P<0.01$; *** $P<0.001$, ND, not detectable (Mann-Whitney test (**A, F**) or two-way ANOVA **(B-E,G,I,J)).** Data are from three (**B**, **C**, **E**, **G, I, J**) or four (**A**, D, **F**) independent experiments (mean and s.e.m.).

Supplementary Figure 2

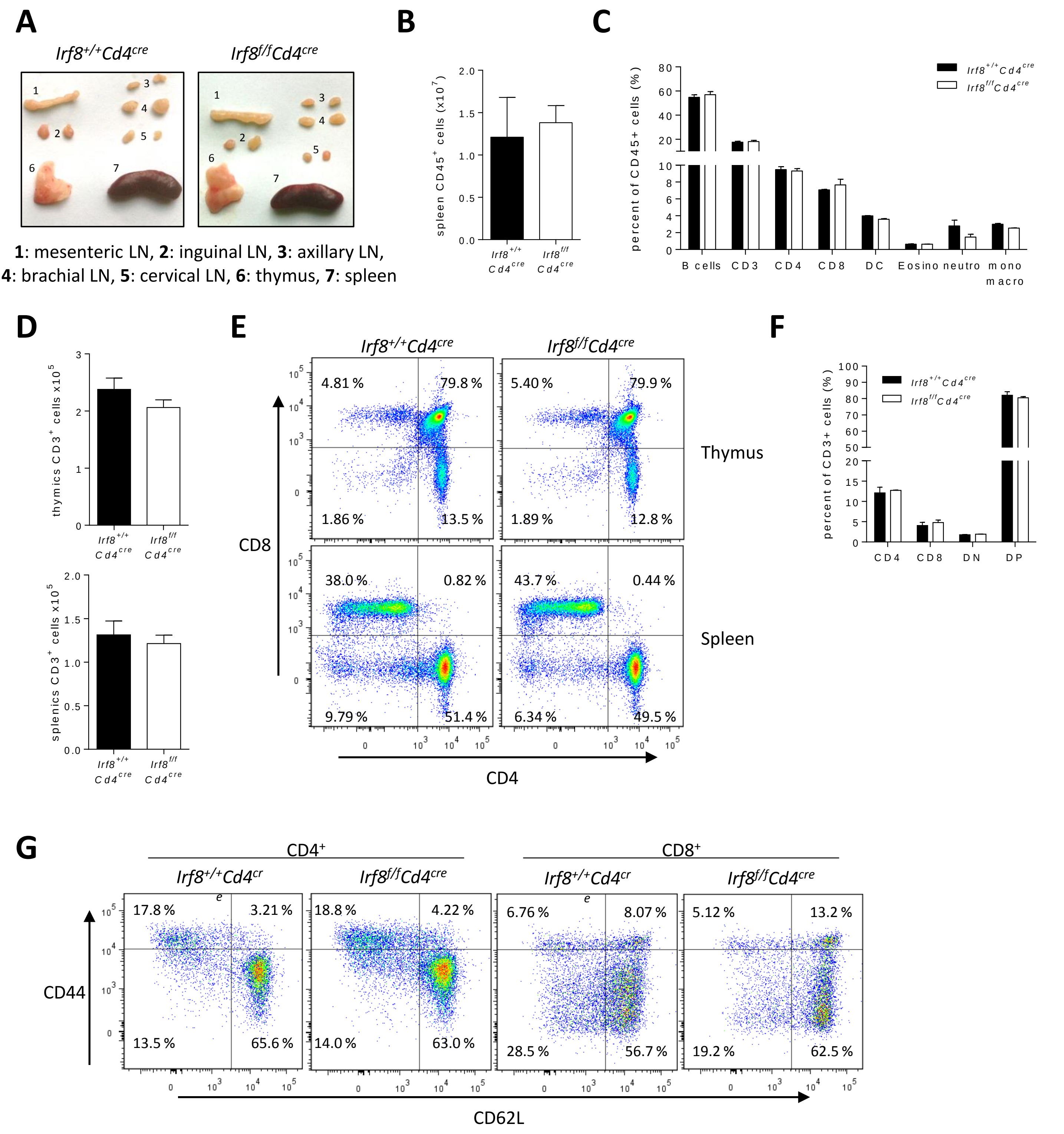

Supplementary Fig.2

A. Lymphoid organs (lymph nodes (LN), spleen and thymus) from *Irf8*$^{+/+}$*Cd4*^{cre} and *Irf8*$^{f/f}$*Cd4*^{cre}

B-C. Absolute number of CD45+ cells (**B**) and frequency of T cells, B cells and myeloid cells (**C**) from *Irf8*$^{+/+}$*Cd4*^{cre} or *Irf8*$^{f/f}$*Cd4*^{cre} mice.

D-F. Absolute number of thymic (top panel) and splenic (bottom panel) CD3+ cells (**D**) and frequencies of CD4+, CD8+, double positive (DP) and double negative (DN) T cells in thymus and spleen (**E-F**) from *Irf8*$^{+/+}$*Cd4*^{cre} or *Irf8*$^{f/f}$*Cd4*^{cre}.

G. Frequencies of splenic CD4+, CD8+ (**G**), active (CD44+) and naïve (CD62L+) T cells from *Irf8*$^{+/+}$*Cd4*^{cre} or *Irf8*$^{f/f}$*Cd4*^{cre}.

2 independent experiments (mean and s.e.m.).

Supplementary Figure 3

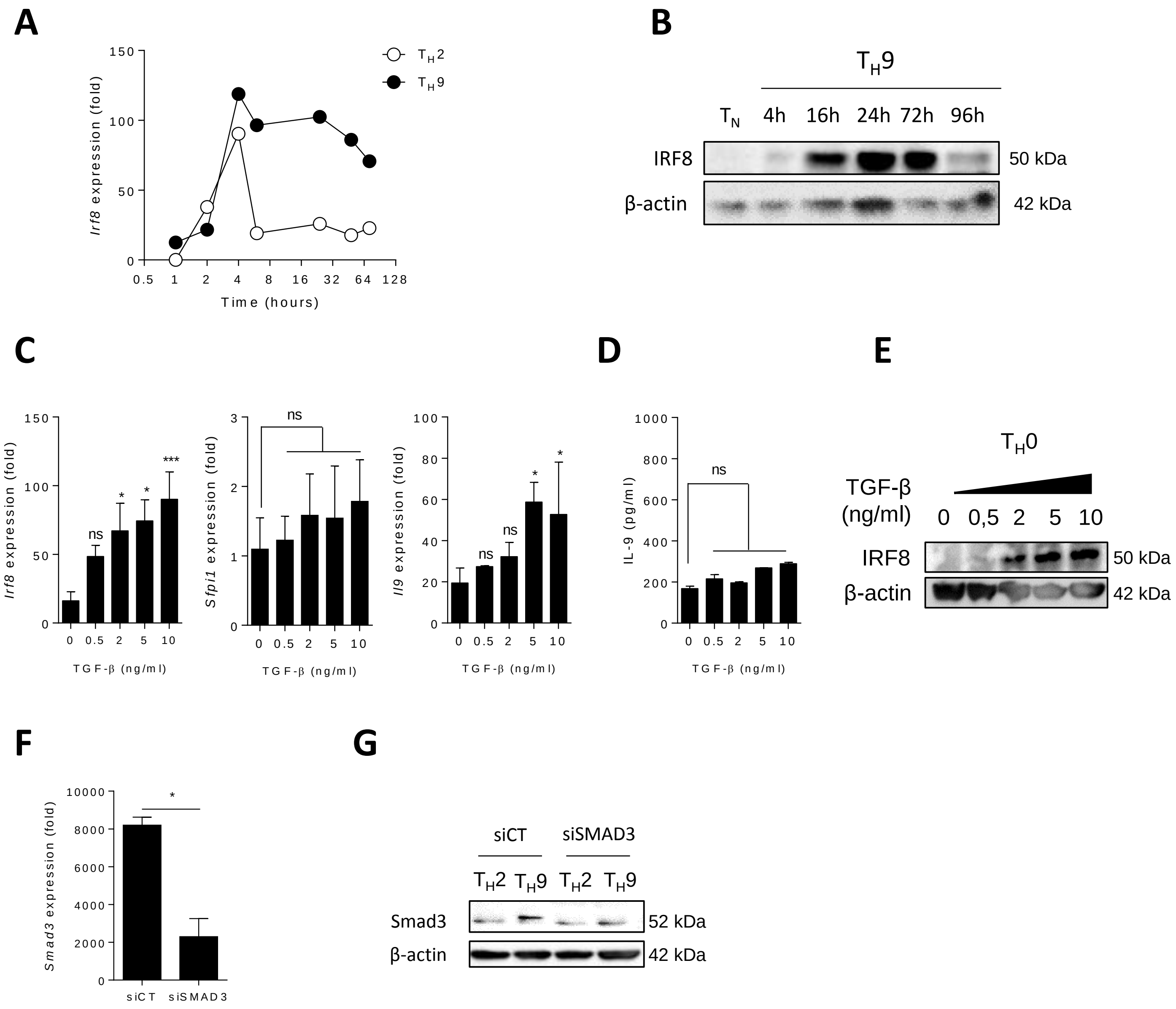

Supplementary Fig.3

A. Kinetic analysis of *Irf8* mRNA expression in WT Th2 and Th9 cells for the indicated time points.

B. Kinetic analysis of IRF8 protein expression by immunoblot in WT Th9 cells for the indicated time points.

C-E. Th0 cells generated by TCR triggering without adding polarizing cytokines were treated with TGF-β increasing dose (0, 0.5, 2, 5 and 10 ng/ml). RT-PCR analysis of *Irf8, Sfpi1* and *il9* mRNA in Th0 cells treated for 24h (**C**). ELISA of IL-9 in supernatant of treated cells after 3 days (**D**). Immunoblot analysis of IRF8 in Th0 cells treated for 24h (**E**).

F-G. RT-PCR analysis of *Smad3* expression in Th9 cells transfected with siRNA sontrol (siCT) or against SMAD3 (siSMAD3) (**F**). Immunoblot analysis of SMAD3 in Th2 and Th9 cells transfected with siCT or siSMAD3 (**G**).

ns, not significant; * $P<0.05$, ** $P<0.01$; *** $P<0.001$ (Mann-Whitney test (**F**) or Kruskal-Wallis test (**C,D)).** Data are from three independent experiments (mean and s.e.m.).

Supplementary Figure 4

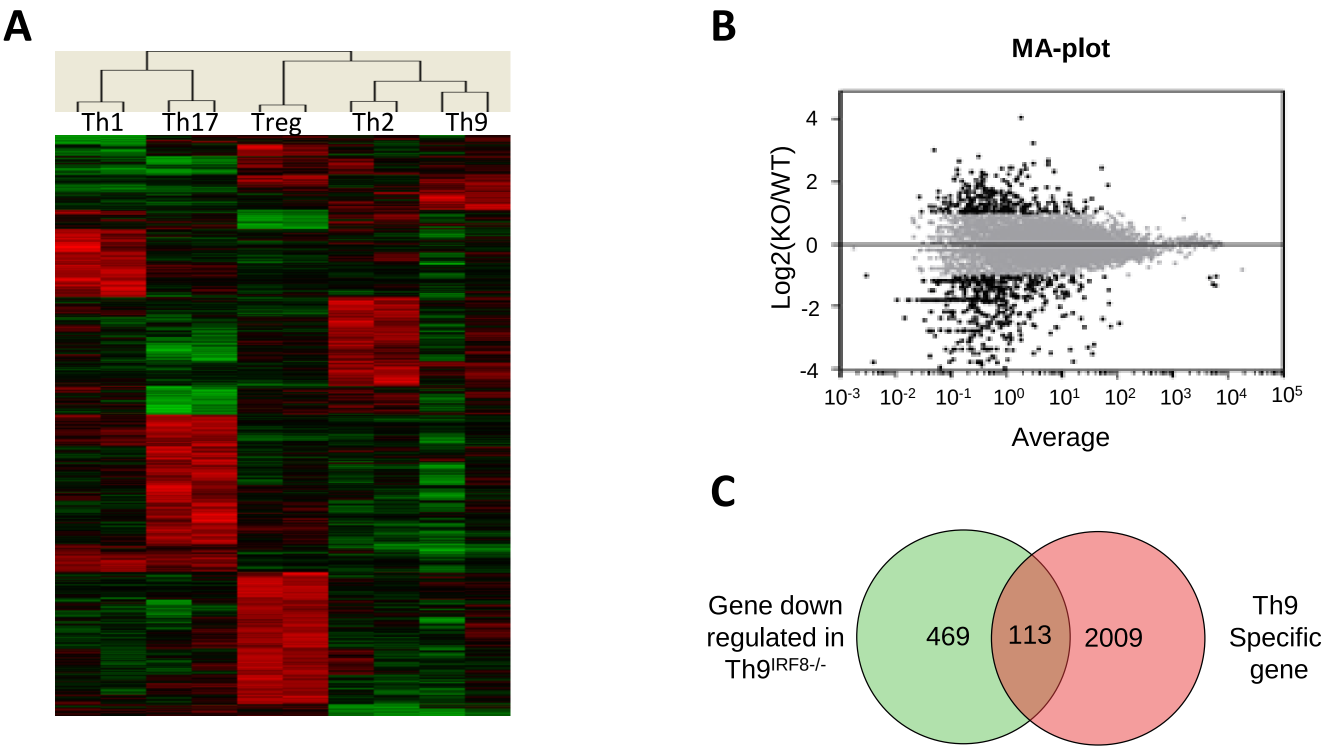

Supplementary Fig.4

A. Heat map displaying RNA sequencing data of WT Th1, Th2, Th9, Treg and Th17 cells differentiated 48h *in vitro*. Heat map defined genes specifically expressed in each T $CD4^{+}$ subsets.

B. MA-plot displaying RNA sequencing data of WT or $IRF8^{-/-}$ Th9 cells after 48h of differentiation. Genes up or downregulated of at least 2 fold in $Th9^{IRF8-/-}$ compare to WT Th9 were labeled in black, genes with similar expression in both $IRF8^{-/-}$ and WT Th9 were labeled in grey.

C. Venn diagram of genes down-regulated in $Th9^{IRF8-/-}$ at 48h (green) and Th9 cells-specific genes defined in **a** (red).

Supplementary Figure 5

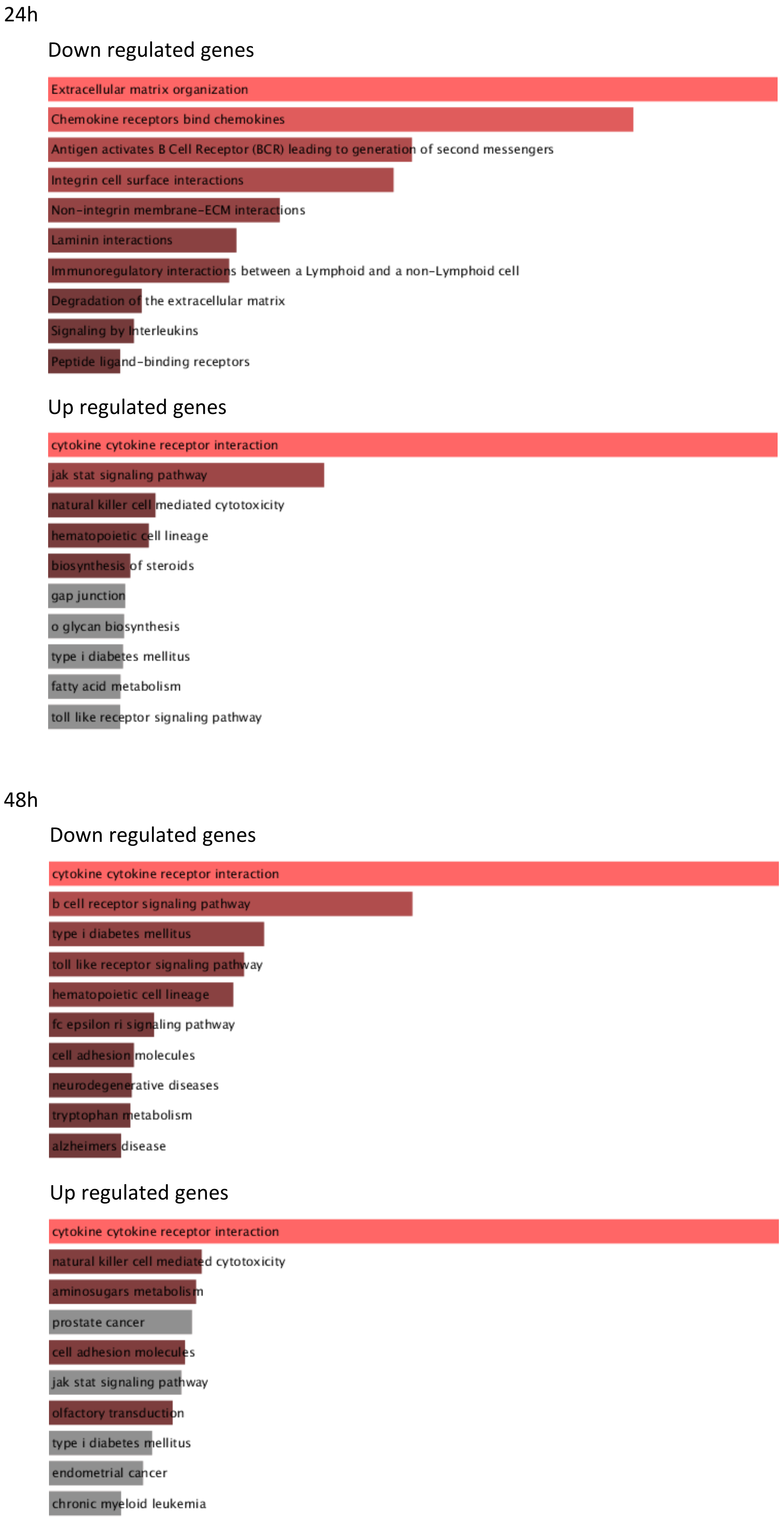

Supplementary Fig.5

Functional gene networks up or down regulated in $Th9^{IRF8-/-}$ at 24h (top panel) or 48h (bottom panel) obtained from Enrichr's web-based tools.

Supplementary Figure 6

A

Database: HOCOMOCOv10 MOUSE
mono meme format

Name	*p*-value	*E*--value	*q*-value	Overlap	Offset	Orientation	sequence
**IRF8** MOUSE.H10MO.D	1.85e-02	7.90e+00	6.31e-01	14	-1	Reverse complement	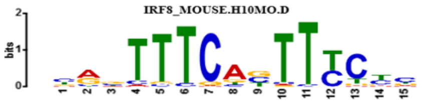
**SPI1** MOUSE.H10MO.A	1.00e-02	4.28e+00	5.61e-01	13	3	Reverse complement	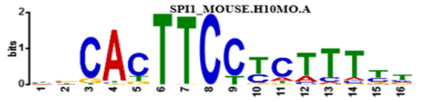
**IRF4** MOUSE.H10MO.C	9.32e-04	3.98e-01	1.57e-01	14	2	Reverse complement	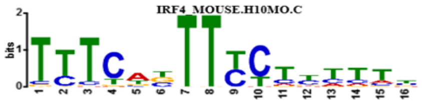

B

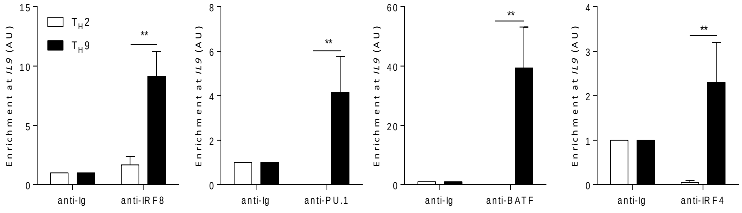

C

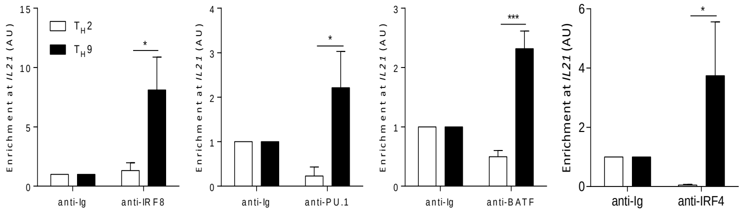

D

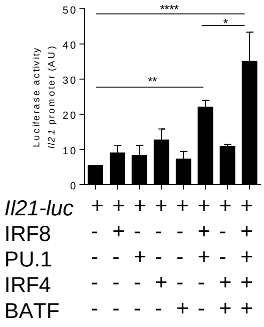

Supplementary Fig.6

A. Analysis of the motif 5'-tbtstbtvtktbtbt-3' using the motif comparison tool Tomtom (http://meme-suite.org/tools/tomtom). This sequence matches with IRF8, IRF4 and PU.1 consensus binding sequence.

B-C. ChIP analysis of the binding of IRF8, BATF, IRF4 and PU.1 to the CNS1 of *Il9* (**B**) or *Il21* (**C**) promoter in Th2 and Th9 cells.

D. Firefly luciferase activity in 3T3 cells given transfection of luciferase reporter constructs for the *Il21* promoter together with various combinations of vectors encoding IRF8, IRF4, BATF and PU.1.

ns, not significant; * P<0.05, ** P<0.01; *** P<0.001 (Two-way ANOVA test (**B**, **C**, **D**)). Data are from three independent experiments (mean and s.e.m.).

Supplementary Figure 7

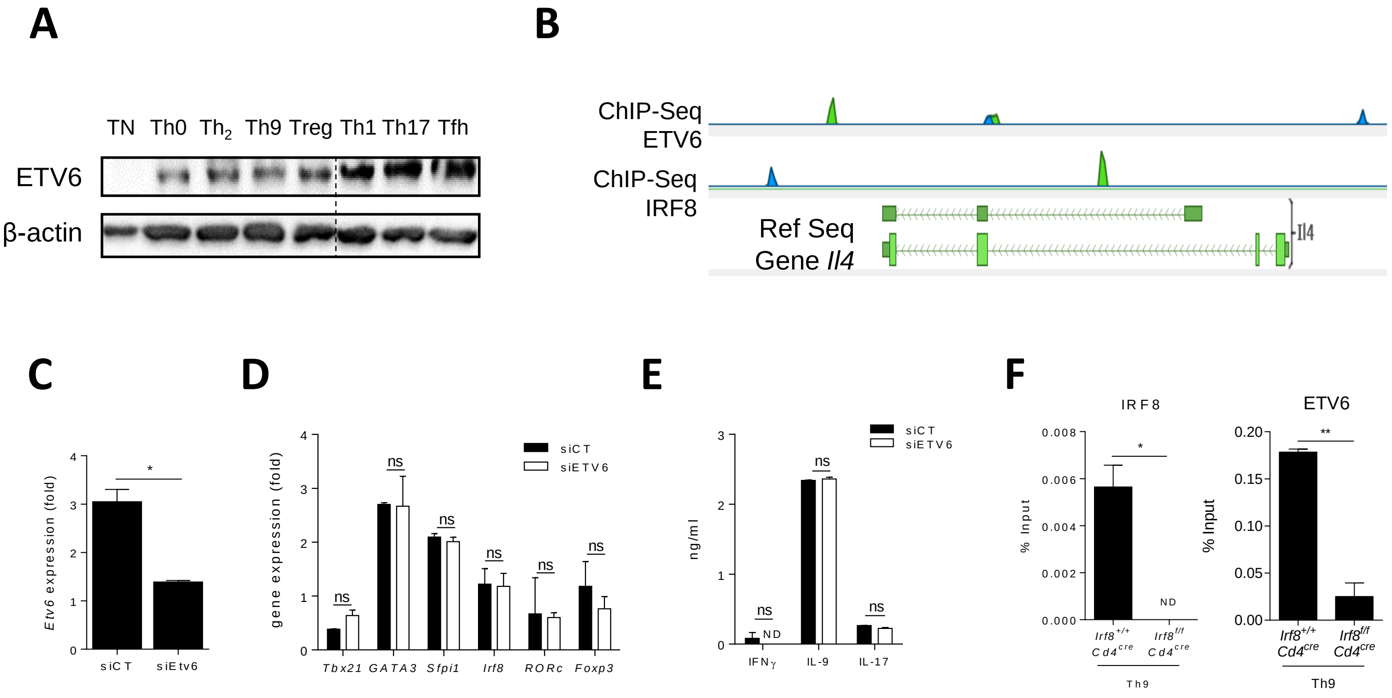

Supplementary Fig.7

A. Immunoblot analysis of ETV6 in naive TCD4+ (TN), Th0, Th1, Th2, Th9, Treg, Th17 and Tfh cells differentiated 24h *in vitro*.

B. Chip-sequencing analysis of the *Il4* gene after Chip-Seq for ETV6 and IRF8 in Th2 cells differentiated for 24h from naive WT CD4+ T cells.

C. RT-PCR analysis of *Etv6* expression in Th9 cells transfected with siRNA sontrol (siCT) or against ETV6 (siETV6).

D. Expression of master regulator genes (*Tbx21*, *GATA3*, *RORc*, *Foxp3*, *Irf8* and *Sfpi1*) in Th9 cells, transfected with siCT or siETV6.

E. Cytokine release (IFNγ, IL-9 and IL-17) by Th9 cells transfected with siCT or siETV6.

F. ChIP analysis of the binding of IRF8 and ETV6 to the HS2 locus of *Il4* in Th9 cells differentiated from $Irf8^{+/+}Cd4^{cre}$ or $Irf8^{f/f}Cd4^{cre}$ cells.

ns, not significant; * $P<0.05$, ** $P<0.01$; *** $P<0.001$, ND, not detectable (Mann-Whitney test(**C,F**) or two-way ANOVA **(D, E)).** Data are from three (**C**) or two (**D-F**) independent experiments (mean and s.e.m.).

Supplementary Figure 8

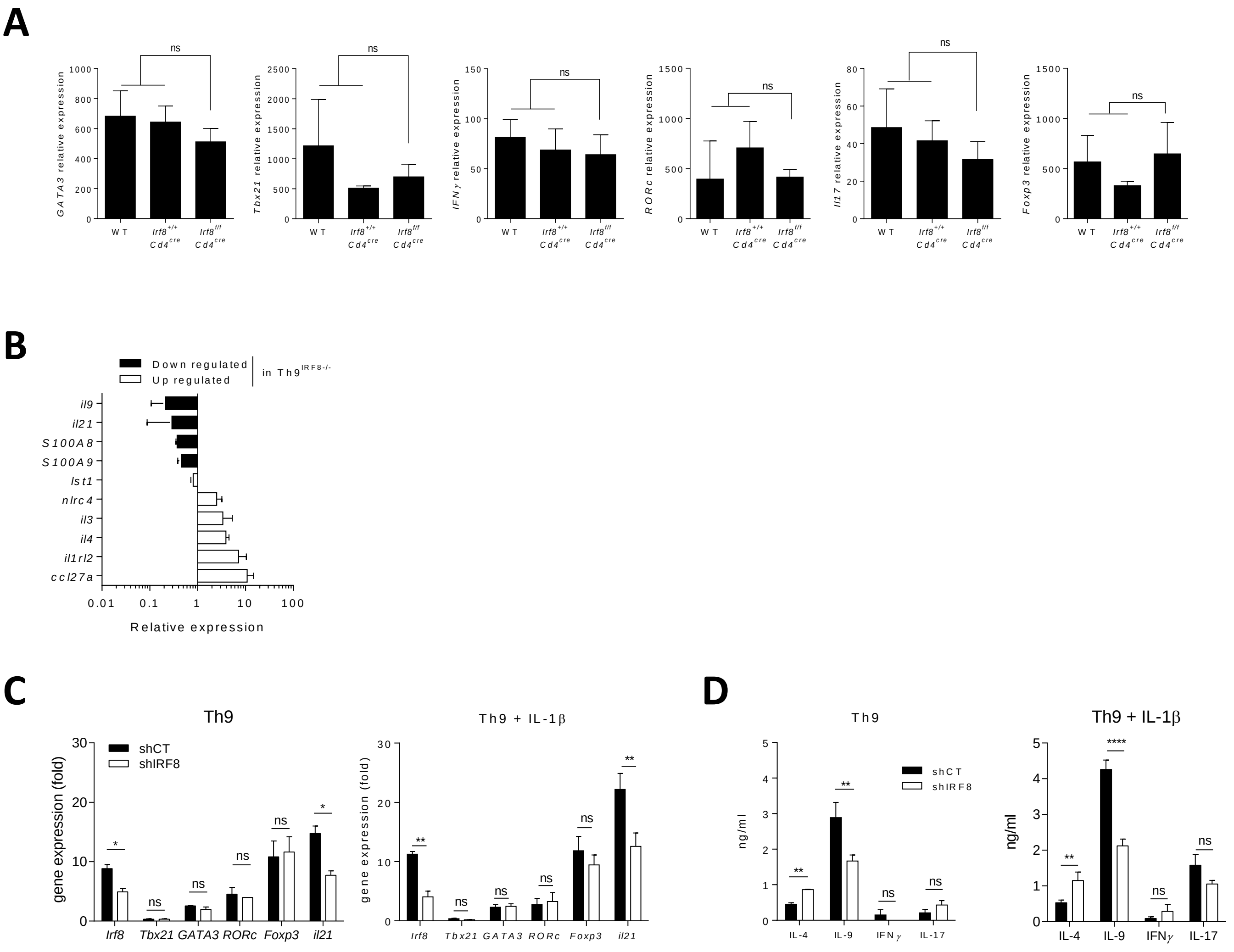

Supplementary Fig.8

A. RT-PCR analysis of Th17 (*RORc*, *Il17*), Treg (*Foxp3*), Th2 (*GATA3*) and Th1 (*Tbx21*, *Ifnγ*) genes expression from tumor infiltrated lymphocytes (TILs) of B16F10 tumor-bearing WT, *Irf8*$^{+/+}$*Cd4*^{cre} or *Irf8*$^{f/f}$*Cd4*^{cre} mice.

B. mRNA expression analysis of *Il9*, *Il21*, *S100A8*, *S100A*, *Il3*, *Ccl27a*, *Il1rl2*, *Lst1* and *Nlrc4* in TILs of B16F10 tumor-bearing *Irf8*$^{+/+}$*Cd4*^{cre} or *Irf8*$^{f/f}$*Cd4*^{cre} mice.

C. mRNA expression analysis of *Irf8*, *Tbx21*, *Gata3*, *Rorc*, *Foxp3* and *Il21*) in Th9 (left panel) or Th9 + IL-1β (right panel) cells infected with shCT or shIRF8.

D. Cytokine release (IFNγ, IL-4, IL-9 and IL-17) by Th9 (left panel) or Th9 + IL-1β (right panel) cells infected with shCT or shIRF8.

ns, not significant; * P<0.05, ** P<0.01; *** P<0.001 (Kruskal-Wallis test (**A**)). Data are from three independent experiments (mean and s.e.m.).

Supplementary Figure 9

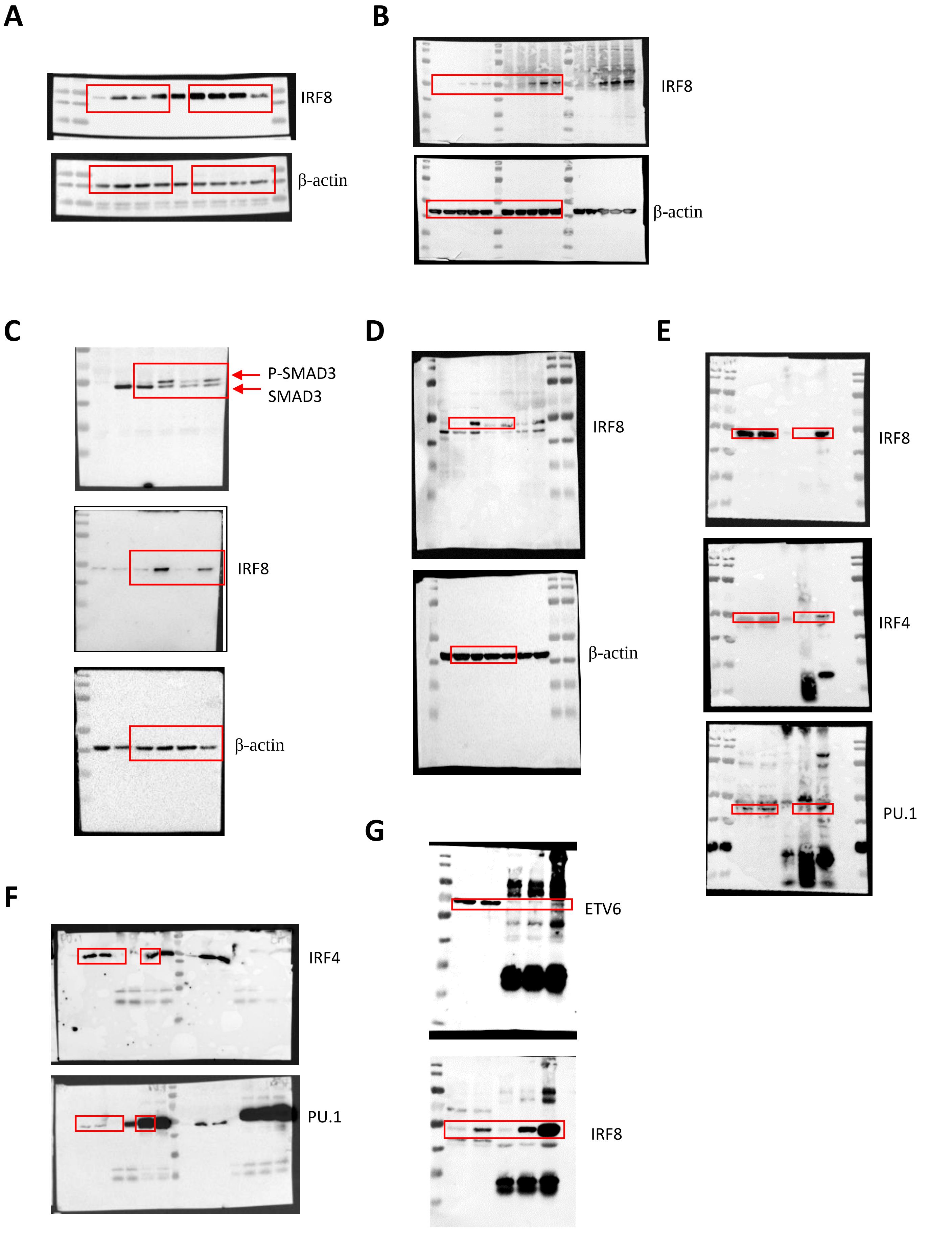

Supplementary Fig.9: Uncropped gel images of western blots

(A) Fig.1A **(B)** Fig.2C **(C)**) Fig.2E **(D)** Fig.2G **(E)** Fig.4C top panel **(F)** Fig.4C bottom panel **(G)** Fig.5C

Supplementary Figure 10

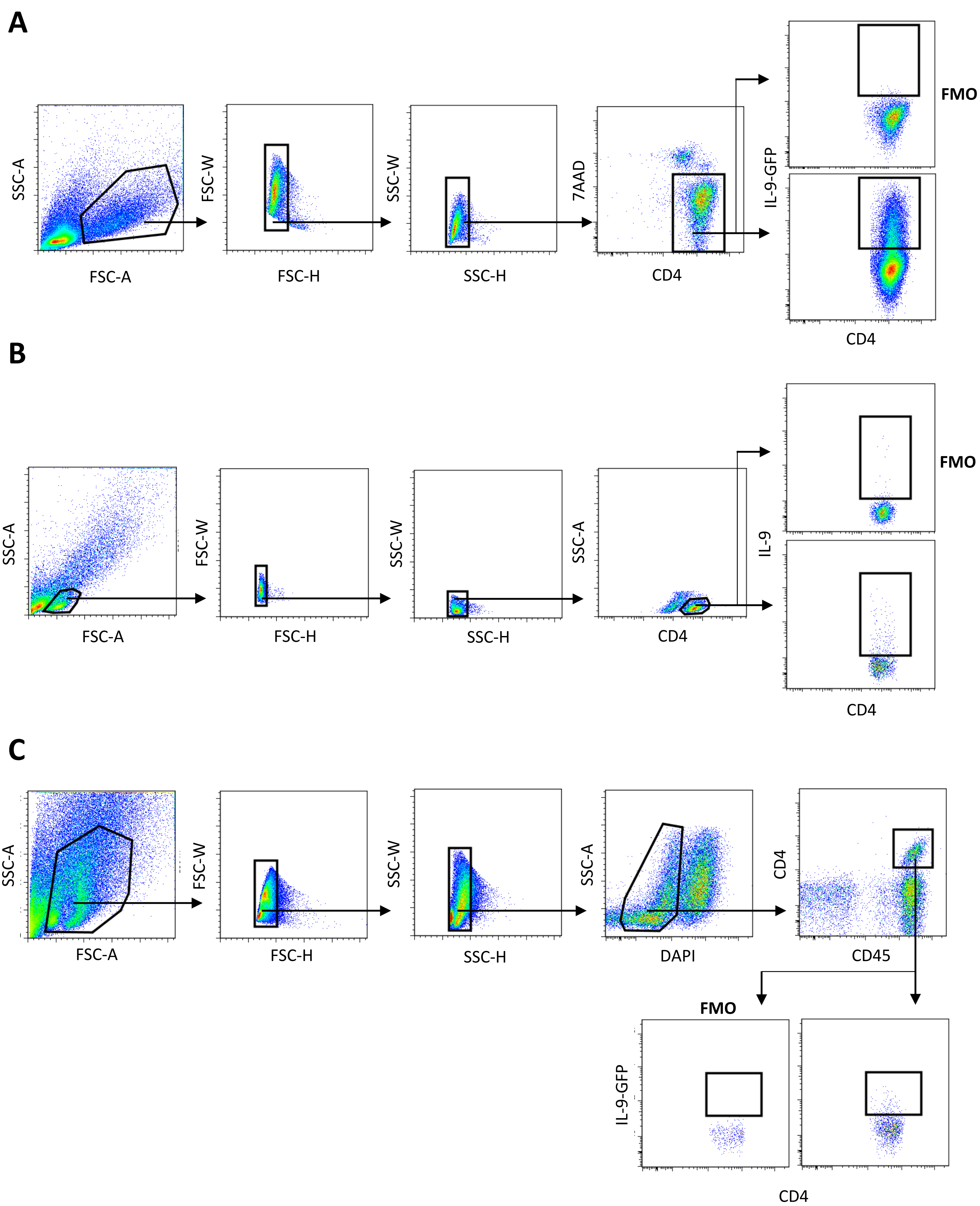

Supplementary Fig.10: FACS sequential gating strategies

A. Fig.1D IL-9-eGFP assessment in Th9 cells differentiated *in vitro*.

B. Fig.1I, IL-9 intracellular staining in Th9 cells differentiated *in vitro*.

C. Fig.2J, IL-9-eGFP assessment in TILs from B16F10 tumour

Supplementary Table 1 : PCR Primer listing

		Forward 5' →3'	Reverse 5' →3'
**PCR:**	*β Actin*	atggaggggaatacagccc	ttctttgcagctccttcgtt
	Ccl27a	tctggggatgaacacagaca	gattgtccacatggaactgc
	Etv6	gatccctttcgctgtgagaca	ggctgcaaacgcaggtgt
	Foxp3	ctcgtctgaaggcagagtca	tggcagagaggtattgaggg
	Gata3	aggatgtccctgctctcctt	gcctgcggactctaccataa
	Ifnγ	tgagctcattgaatgcttgg	acagcaaggcgaaaaaggat
	Il17a	tgagcttcccagatcacaga	tccagaaggccctcagacta
	Il21	aaaacaggcaaaagctgcat	tgacattgttgaacagctgaaa
	Il3	ttccacgaatttggacaggt	atagggaagctcccagaacc
	Il4	tgaacgaggtcacaggagaa	cgagctcactctctgtggtg
	Il9	aacagtccctccctgtagca	aaggatgatccaccgtcaaa
	Irf4	caaagcacagagtcacctgg	tgcaagctctttgacacaca
	Irf8	ttcaaggcaggtggtggt	ggatatgccgcctatgacac
	Lst1	agtcccaggctcccatttag	ttgaccttcaggcctgttct
	Nlrc4	cggcctgcaacctctttctt	tgggccaaaacattcaggtct
	Rorc	ggtgataaccccgtagtgga	ctgcaaagaagacccacacc
	S100a8	ccaattctctgaacaagttttcg	tcaccatgccctctacaaga
	S100a9	gtccaggtcctccatgatgt	tcagacaaatggtggaagca
	Sfpi1	tgcagctctgtgaagtggtt	agcgatggagaaagccatag
	Smad3	aggaggagaagtggtgcga	ccagtgacctggggatggtaat
	Tbx-21	atcctgtaatggcttgtggg	tcaaccagcaccagacagag
**ChIP:**	pIl9_CNS1	tgatacccagtgcccactttt	tccaaggatcctcaaggcca
	pl21_CNS1	ttggctaggtgtacgtgtgc	agtgtcaggaggcaccattag
	pIl4_HS2	gctctgtctgcatcaagac<u>gc</u>	ctaggggaatggggtggaaca
	pIrf8_-1275	ctgcatacgcttataaggtgcc	gtgccaccatagtccagctc
**Cloning:**	pGl3_pIl9	taacgcgtaacgtattgactatcttctg	tactcgagccttggtctagaatagctta
	pGl3_pIRF8	taacgcgttttgatatttgtaagtagga	agttctcgagatttctcttcattatgaagt

Supplementary Table 2 : Antibodies listing and their use

Antibodies were use for : Immunoblot (IB), Immunoprecipitation (IP), Chromatine IP (ChIP) or Proximity Ligation Assay (PLA)

Target	Clone	Isotype	Provider	Use			
				**IB**	**ChIP**	**IP**	**PLA**
β-actin	AC-15	Mousé, IgG1	Sigma-Aldrich	X			
BATF	D7C5	Rabbit, IgG	Cell signaling	X	X	X	
BATF	ww8	Mouse, IgG1	santa cruz				X
ETV6 / TEL	polyclonal	Rabbit, IgG	abcam	X	X		X
H3K9Ac	polyclonal	Rabbit, IgG	abcam		X		
H3K9me3	polyclonal	Rabbit, IgG	abcam		X		
Ig Goat			Dako		X	X	X
Ig rabbit			Dako		X	X	X
IRF4	polycolnal (M-17)	Goat, IgG	santa cruz	X	X		X
IRF8 / ICSBP	D20D8	Rabbit, IgG	Cell signaling	X	X	X	X
PU.1 / Spi-1	polycolnal (T-21)	Rabbit, IgG	santa cruz	X	X	X	X
PU.1 / Spi-1	polycolnal (D-19)	Goat, IgG	santa cruz				X
pSMAD3 (S423/425)	C25A9	Rabbit, IgG	Cell signaling	X			
SMAD3	C67H9	Rabbit, IgG	Cell signaling	X			

Supplementary Table 3 : antibodies for cytometry

Target	Fluorochrome	Clone	Isotype	Dilution	Provider
CD4	fitc	RM4-5	Rat, IgG2a	1/100	BD Biosciences
CD4	APC-Vio770	GK1.5	Rat, IgG2b	1/10	Miltenyi Biotech
CD45	pe-cy7	30F11	Rat, IgG2b	1/100	BD Biosciences
CD45	percp-cy5.5	30F11	Rat, IgG2b	1/100	BD Biosciences
IL-9	APC	RM9A4	Rat, IgG1	1/100	Biolegend
